# Supplementary material for: Simple Route to Synthesize Fully Conjugated Ladder Isomer Copolymers with Carbazole Units
Source: Polymers (Basel). 2019 Oct 7;11(10):1619. doi: 10.3390/polym11101619 (PMC6835825; doi:10.3390/polym11101619)
Supplement: Supplementary file 1 [file polymers-11-01619-s001.pdf]

## Supplementary Files

# Simple Route to Synthesize Fully Conjugated Ladder Isomer Copolymers with Carbazole Units

**Shuang Chen[1], Feng Liu[1,2,*], Chao Wang[1], Jinghui Shen[1], Yonggang Wu[1,*]**

[1] College of Chemistry and Environmental Science, Hebei University, Baoding, 071002, P.R. China. E-mail: wuyonggang@hbu.edu.cn

[2] College of Physics Science and Technology, Hebei University, Baoding 071002, P. R. China. Postdoctoral of Hebei University. E-mail: liufeng@hbu.edu.cn

* Correspondence: liufeng@hbu.edu.cn; wuyonggang@hbu.edu.cn; Tel.: +86-158-3121-6174

## Materials and Instruments

Commercial chemicals were used without further purification. Tetrahydrofuran (THF), and dichloromethane (DCM) were distilled by a standard process before using. The reactions were monitored by thin layer chromatography (TLC) with silica gel 60 F254 (Merck, 0.2 mm). $^{1}H$ and $^{13}C$ NMR data were acquired on a Bruker AV600 spectrometer. The electrochemical behavior was recorded by cyclic voltammetry (Holland, Ivium Plus II) with a standard three-electrode electrochemical cell. A glassy carbon working electrode, a Pt wire counter electrode, and an $Ag/Ag^{+}$ (0.01 M in $CH_3CN$) reference electrode. UV-visible absorption spectra were obtained on a Shimadzu UV-visible spectrometer model UV-2550. Fluorescence spectra were investigated by a Shimadzu RF-5301PC fluorescence spectrophotometer. MALDI-TOF analyses were obtained by Bruker Daltonics Inc Autoflex III. Thermal gravimetric analysis (TGA) and differential scanning calorimetry (DSC) measurements were carried out under nitrogen on Perkin-Elmer Pyris 6 TGA (heating rate of 10 $^{o}$C/min) and Perkin-Elmer Diamond DSC instruments (scanning rate of 10 $^{o}$C/min), respectively, to record TGA and DSC curves.

## Experimental Section

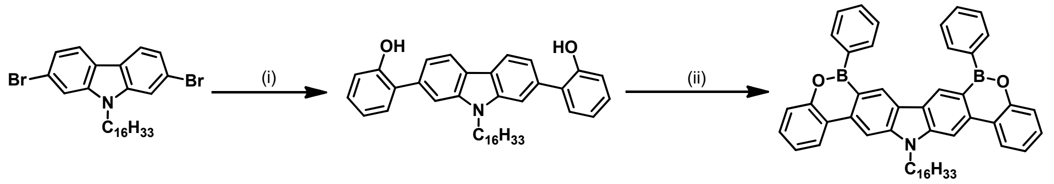


a1 a2 S1

Scheme S1. The synthetic routes to compound S1. (i) $NaHCO_3$, THF/$H_2O$, $Pd(PPh_3)_4$, reflux, 12h. (ii) TEA, *o*-DCB, reflux, 12h.

## Compound a2

A mixture of compound a1 (0.30 g, 0.55 mmol), 2-hydroxyphenylboronic acid (0.60 g， 0.93 mmol), $NaHCO_3$ (0.35 g), THF (25 mL) and $H_2O$ (4 mL) was degassed, $Pd(PPh_3)_4$ (12 mg, 0.01 mmol) was added under a nitrogen atmosphere. The mixture was heated at reflux and stirred under nitrogen for 12 h. After cooling, water and $CH_2Cl_2$ (200 mL) were added, the organic layer was separated and dried over $Na_2SO_4$. After removal of the solvent, the residue was chromatographically purified on silica gel eluting with petroleum ether/dichloromethane (v/v=1/3) to afford compound a3 (0.26 g, 83 %). $^{1}H$ NMR (600 MHz, $CDCl_3$, ppm): δ = 8.15 (d, J = 7.9 Hz, 2H), 7.42 (s, 2H), 7.31 (dd, J = 7.9, 1.5 Hz, 2H), 7.28 (dd, J = 7.9, 1.2 Hz, 2H), 7.25 (td, J = 7.9,

1.5 Hz, 2H), 6.98 (t, J = 7.1 Hz, 4H), 5.41 (s, 2H),4.25 (t, J = 7.3 Hz, 2H), 1.84 – 1.78 (m, 2H), 1.33 – 1.28 (m, 2H), 1.25 –1.20 (m, 4H), 1.15 (d, J = 17.8 Hz, 21H), 0.80 (t, J = 7.0 Hz, 3H); $^{13}$C NMR (150MHz, $CDCl_3$, ppm): δ = 151.60, 140.52, 133.70, 129.43, 128.16, 127.86, 121.18, 120.38, 119.79, 118.95, 114.77, 108.37,42.29, 30.90, 28.77, 28.23, 27.98, 26.33, 21.66, 13.09; MS (MALDI-FTICR): m/z: calcd for $C_{40}H_{49}NO_2$ : 575.8200 $[M]^+$; found: 576.3757.

## Compound S1

A mixture of compound a2 (0.20g, 0.35mmol), phenylboron dichloride (0.17g, 1.05mmol), Triethylamine (8 drops) and 1,2-dichlorobenzene (10 mL) were heated at reflux and stirred under nitrogen for 6 h. After removal of the solvent, the residue was chromatographically purified on silica gel eluting with petroleum ether/dichloromethane (v/v=1/1) to afford compound S1 (0.25 g, 96%). $^1$H NMR (600 MHz, $CDCl_3$, ppm): δ= 9.03 (s, 2H), 8.33 (d, J =7.8 Hz, 2H), 8.14 (d, J = 5.6 Hz, 4H), 8.11 (s, 2H), 7.60 (d, J = 5.9 Hz, 6H), 7.55 (d, J = 8.0Hz, 2H), 7.46 (t, J = 7.4 Hz, 2H), 7.34 (t, J = 7.4 Hz, 2H), 4.40 (t, J = 6.9 Hz, 2H), 2.02~1.95(m, 2H), 1.50~1.45 (m, 2H), 1.42~1.38 (m, 2H), 1.26 (d, J = 7.0 Hz, 8H), 1.22 (d, J = 11.0 Hz, 14H), 0.87 (t, J = 7.0 Hz, 3H); $^{13}$C NMR (150 MHz, $CDCl_3$, ppm): δ = 151.51, 145.06, 138.03, 134.53, 130.32, 130.05, 128.91, 128.16, 123.65, 123.41, 123.18, 123.08, 120.66,100.74, 43.08, 31.93, 29.66, 29.54, 29.40, 29.36, 28.66, 27.30, 22.70, 14.12; MS (MALDI-FTICR): m/z: calcd for $C_{52}H_{55}B_2NO_2$ : 747.6200; found: 747.4420.

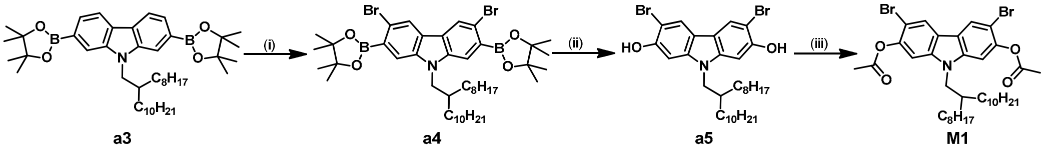


Scheme S2. The synthetic routes to compound M1. (i) NBS, DMF, 110 °, 30 min. (ii) acetone, $H_2O_2$, NaOH. (iii) DCC, DMAP, THF/$CH_3COOH$, 45 °C, 12h.

## Compound a4

A mixture of compound a3 (0.70 g， 1.00 mmol), NBS (0.39 g，2.20 mmol) and 25 mL DMF was heated at 110 °C and stirred under nitrogen for 30 min. After cooling, water and $CH_2Cl_2$ (200 mL) were added, the organic layer was separated and dried over $Na_2SO_4$. After removal of the solvent, the residue was chromatographically purified on silica gel eluting with petroleum ether/dichloromethane (v/v=1/1) to afford compound a4 (0.68 g, 80 %). $^1$H NMR (600 MHz, $CDCl_3$, ppm): δ = 8.18 (s, 2H), 7.66 (s,2H), 4.16 (d, J = 7.1 Hz, 2H), 2.06 (s, 1H), 1.32 (d, J = 62.5 Hz, 56H), 0.87 (q, J = 5.1, 4.6 Hz,6H); $^{13}$C NMR (100 MHz, $CDCl_3$, ppm): δ =139.76, 124.77, 124.67, 117.21, 116.90, 84.30,37.95, 31.92, 31.88, 31.85, 29.89, 29.62, 29.53, 29.34, 29.27, 26.52, 24.84, 22.68, 22.66,14.11; MS (MALDI-TOF): m/z: calcd for $C_{44}H_{69}B_2Br_2NO_4$: 857.5 $[M]^+$; found: 856.9.

## Compound a5

Compound a4 (0.50 g，0.58 mmol) was dissolved in acetone (15 ml) and $H_2O_2$ (1.5 mL) was added in the mixture. Then NaOH aqueous solution (1.7 M, 1.5 mL) was added and stirred at room temperature for 15 min. After that, water and $CH_2Cl_2$ (200 mL) were added, the organic layer was separated and dried over $Na_2SO_4$. After removal of the solvent, the residue was chromatographically purified on silica gel eluting with petroleum ether/dichloromethane (v/v=1/1)

to afford compound a5 (0.32 g, 86 %). $^1$H NMR (600 MHz, $CDCl_3$, ppm): δ = 7.94 (s,2H), 6.94 (s, 2H), 5.63 (s, 2H), 3.92 (d, J = 7.5 Hz, 2H), 2.03 (s, 1H), 1.22 (d, J = 18.0 Hz,32H), 0.87 (dt, J = 6.8, 3.0 Hz, 6H); $^{13}$C NMR (100 MHz, $CDCl_3$, ppm): δ = 150.16, 142.15,122.27, 117.22, 102.10, 95.99, 95.97, 48.23, 37.43, 31.92, 31.85, 31.82, 29.91, 29.59, 29.58,29.50, 29.45, 29.32, 29.23, 26.54, 22.69, 22.65, 14.12; MS (MALDI-TOF): m/z: calcd for$C_{32}H_{47}Br_2NO_2$: 637.5 $[M]^+$; found: 636.8.

## Compound M1

Compound a5 was dissolved in THF (25 mL) with 0.1 mL of $CH_3COOH$. DCC (0.26 g，1.26 mmol) and DMAP (0.06 g，0.50 mmol) were added in batches. Then the mixture was heated at 45 °C for 12 hours. After cooling, removal of the solvent, the residue was chromatographically purified on silica gel eluting with petroleum ether/dichloromethane (v/v=1/3) to afford compound M1 (0.26 g, 83 %). $^1$H NMR (600 MHz, $CDCl_3$, ppm): δ = 8.14 (s, 2H), 7.09 (s, 2H), 3.95 (s, 2H),2.42 (s, 6H), 1.99 (s, 1H), 1.20 (s, 32H), 0.88 (s, 6H); $^{13}$C NMR (100 MHz, $CDCl_3$, ppm): δ =168.88, 146.32, 140.93, 124.30, 120.81, 106.81, 104.46, 104.43, 37.65, 31.92, 31.87, 31.73,29.86, 29.62, 29.57, 29.54, 29.48, 29.33, 29.22, 26.42, 22.70, 22.65, 20.96, 20.92, 14.12; MS(MALDI- FTICR): m/z: calcd for $C_{36}H_{51}Br_2NO_4$: 721.6000 $[M]^+$; found: 721.2158.

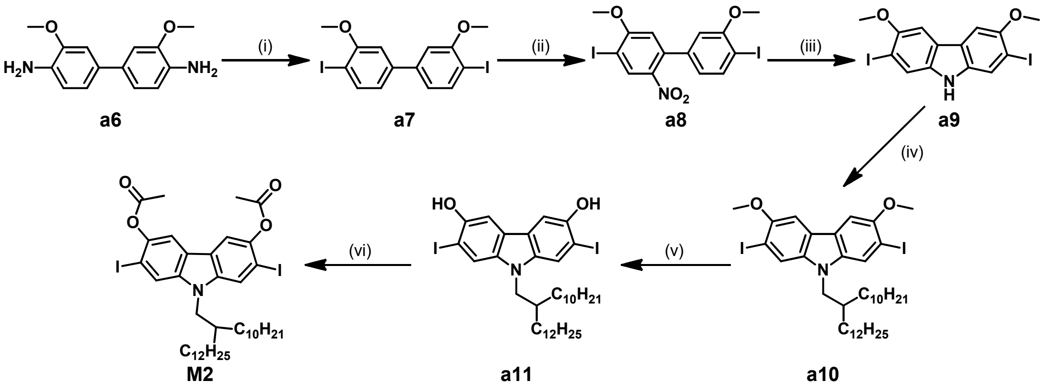


Scheme S3. The synthetic routes to compound M2. (i) HCl, $NaNO_2$, rt, 30 min, KI, rt, 12h. (ii) $HNO_3$, $CH_2Cl_2$, 70 °C. (iii) triethyl phosphite, *o*-DCB (6 mL) , reflux, 12h. (iv) NaH, DMF, 30 °C, 4 h. (v) $BBr_3$, $CH_2Cl_2$, 30 °C, 2 h. (vi) DCC, DMAP, THF/$CH_3COOH$, 45 °C, 12h.

## Compound a7

Compound a6 was dissolved in HCl aqueous solution and $NaNO_2$ (6.20 g，89.86 mmol) was slowly added into the mixture. If some suspended matter were occurred in the solution, more HCl aqueous solution was needed to make the solution clarification. Then the mixture was stirred at room temperature for 30 min. KI (17.00 g，0.10 mol) was added and stirred at room temperature for 12 hours. After cooling, water and $CH_2Cl_2$ (200 mL) were added, the organic layer was separated and dried over $Na_2SO_4$. After removal of the solvent, the residue was chromatographically purified on silica gel eluting with petroleum ether to afford compound a7 (2.50 g, 26 %). $^1$H NMR (600 MHz, $CDCl_3$, ppm): δ =7.82 (d, J = 8.0 Hz, 2H), 6.96 (d, J = 1.5 Hz, 2H), 6.90 (dd, J = 8.0, 1.7 Hz, 2H), 3.95 (s, 6H);13C NMR (150 MHz,CDCl3, ppm): δ = 158.55, 142.42, 139.78, 121.30, 109.83, 85.43, 56.47.

## Compound a8

Compound (2.50 g，5.36 mmol) was dissolved in $CH_2Cl_2$ (20 mL). Dilute $HNO_3$ solution was

added and the reaction was heated at 70 ºC. The reaction was detected by TCL until the reaction finished. After cooling, water and $CH_2Cl_2$ (200 mL) were added, the organic layer was separated and dried over $Na_2SO_4$. After removal of the solvent, the residue was chromatographically purified on silica gel eluting with petroleum ether/dichloromethane (v/v=3/1) to afford compound a8 (2.20 g, 81 %). $^1$H NMR (600 MHz, $CDCl_3$, ppm): δ = 8.44 (s, 1H), 7.81 (d, J = 7.9 Hz, 1H), 6.69 (d, J = 2.7 Hz,2H), 6.65 (dd, J = 7.9, 1.7 Hz, 1H), 3.92 (d, J = 56.4 Hz, 6H);$^{13}$C NMR (150 MHz, $CDCl_3$, ppm): δ = 161.20, 158.23, 142.06, 139.70, 139.32, 138.47, 135.96, 121.73, 112.59, 110.43,86.36, 84.16, 57.16, 56.49.

## Compound a9

A mixture of compound a8 (0.50 g，0.98 mmol), triethyl phosphite (1.6 mL) and *o*-DCB (6 mL) was degassed. The mixture was heated at reflux and stirred under nitrogen overnight. After cooling, water and $CH_2Cl_2$ (200 mL) were added, the organic layer was separated and dried over $Na_2SO_4$. After removal of the solvent, the residue was chromatographically purified on silica gel eluting with petroleum ether/dichloromethane (v/v=10/9) to afford compound a9 (0.10 g, 21 %). $^1$H NMR (600 MHz, $CDCl_3$, ppm): δ = 7.84 (s, 2H), 7.71 (s, 1H), 7.43 (s, 2H),3.99 (s, 6H); $^{13}$C NMR (150 MHz, $CDCl_3$, ppm): δ = 152.09, 135.91, 123.77, 121.58, 101.61,85.08, 57.12.

## Compound a10

A mixture of compound a9 (0.10 g，0.21 mmol), 11-(bromomethyl)tricosane (0.17g，0.42mmol) and NaH (0.07 g) were dissolved in DMF (25 mL). The mixture was heated at 30 ºC and stirred under nitrogen for 4 h. After cooling, water and $CH_2Cl_2$ (200 mL) were added, the organic layer was separated and dried over $Na_2SO_4$. After removal of the solvent, the residue was chromatographically purified on silica gel eluting with petroleum ether to afford compound a10 (0.16 g, 94 %). $^1$H NMR (600 MHz, $CDCl_3$, ppm): δ = 7.75 (s, 2H), 7.45 (s, 2H),3.99 (s, 8H), 2.05 (s, 1H), 1.24 (d, J = 11.5 Hz, 40H), 0.88 (t, J = 6.8 Hz, 6H); $^{13}$C NMR (75MHz, $CDCl_3$, ppm): δ = 151.42, 137.18, 122.68, 119.96, 101.58, 84.92, 57.13, 47.83, 37.74,31.96, 31.95, 31.60, 29.96, 29.71, 29.69, 29.66, 29.62, 29.40, 29.37, 26.36, 22.73, 14.17; MS(MALDI-FTICR): m/z: calcd for $C_{36}H_{51}I_2NO_4$: 815.6000 $[M]^+$; found: 815.2624.

## Compound a11

A mixture of compound a10 (0.14 g，0.17 mmol) and $BBr_3$ (1.5 mL) were dissolved in $CH_2Cl_2$ (20 mL). The mixture was heated at 30 ºC and stirred for 2 h. After cooling, water and $CH_2Cl_2$ (200 mL) were added, the organic layer was separated and dried over $Na_2SO_4$. After removal of the solvent, the residue was chromatographically purified on silica gel eluting with dichloromethane to afford compound a11 (0.10 g, 74 %). $^1$H NMR (600 MHz, $CDCl_3$, ppm): δ = 7.59(d, J = 2.0 Hz, 4H), 5.08 (s, 2H), 3.96 (d, J = 7.5 Hz, 2H), 2.02 (s, 1H), 1.24 (d, J = 10.5 Hz, 40H), 0.88 (t, J = 6.8 Hz, 6H); $^{13}$C NMR (100 MHz, $CDCl_3$, ppm): δ = 147.54, 137.53, 123.69,118.04, 84.80, 47.94, 37.59, 31.95, 31.93, 31.65, 29.94, 29.69, 29.67, 29.65, 29.60, 29.38,29.35, 26.38, 22.71, 14.14; MS (MALDI- FTICR): m/z: calcd for $C_{36}H_{55}I_2NO_2$: 787.6400 $[M]^+$; found: 786.2234.

## Compound M2

Compound a11 (0.09 g，0.11 mmol) was dissolved in THF (25 mL) with 0.1 mL of

$CH_3COOH$. DCC (0.14 g，0.68 mmol) and DMAP (0.03 g，0.27 mmol) were added in batches. Then the mixture was heated at 45 °C for 12 hours. After cooling, removal of the solvent, the residue was chromatographically purified on silica gel eluting with petroleum ether/dichloromethane (v/v=1/1) to afford compound M2 (0.09 g, 96 %). $^1$H NMR (600 MHz, $CDCl_3$, ppm): δ = 7.77 (s, 2H), 7.71 (s, 2H), 4.03 (d, J =7.4 Hz, 2H), 2.42 (s, 6H), 2.04 (s, 1H), 1.25 (s, 40H), 0.88 (t, J = 6.8 Hz, 6H); $^{13}$C NMR (75MHz, $CDCl_3$, ppm): δ = 169.59, 143.63, 140.21, 122.83, 119.46, 113.98, 88.26, 53.45, 48.06,37.73, 31.95, 31.55, 29.96, 29.66, 29.40, 29.38, 26.37, 22.73, 21.37, 14.16; MS (MALDI-FTICR):m/z: calcd for $C_{40}H_{59}I_2NO_4$: 871.7100 $[M]^+$; found: 870.2445.

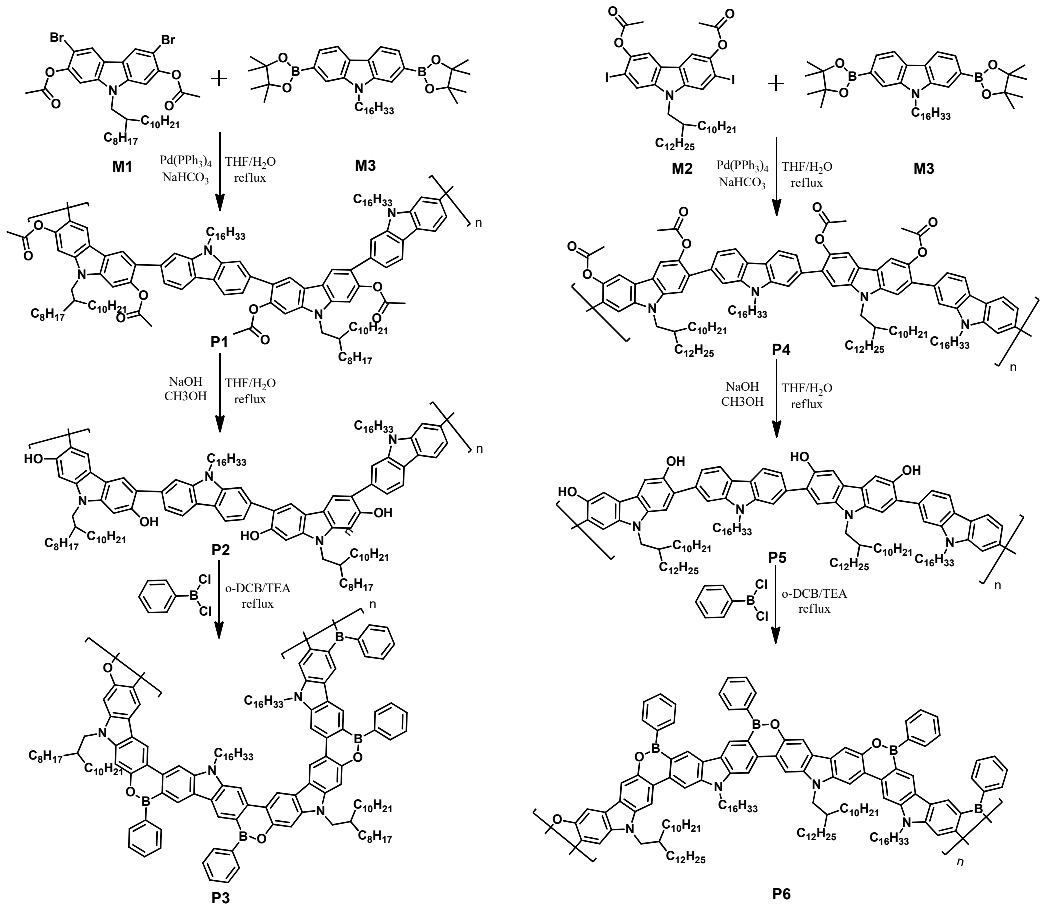


**Scheme S4.** The synthetic routes to polymer P3 and P6

## Polymer P1

A mixture of compound M1 (0.25 g , 0.35 mmol), 2,7-bis(4,4,5,5-tetramethyl-1,3,2-dioxaborolan-2-yl)-9-hexadecyl-9H-carbazole M3 (0.23 g, 0.35 mmol), $NaHCO_3$ (0.35 g), THF (25 mL) and $H_2O$ (4 mL) was degassed, $Pd(PPh_3)_4$ (12.80 mg, 0.01 mmol) was added under a nitrogen atmosphere. The mixture was heated by microwave reactor at reflux and stirred under nitrogen for 4 h. The solution was then cooled to room temperature, precipitated in methanol, and filtered. The solids were washed three times with methanol and

petroleum ether, respectively. Then dried to yield the polymer product P1 (0.31 g, 95%).

**Polymer P2**

A mixture of polymer P1 (0.25 g), NaOH (0.50 g), $H_2O$ (2 mL), $CH_3OH$ (3 mL) and THF (20 mL) were heated at reflux and stirred under nitrogen for 3 h. After cooling to the room temperature, the PH of solution was adjusted to 4~5. The solution was then cooled to room temperature, precipitated in methanol, and filtered. The solids were washed three times with methanol and petroleum ether, respectively. Then dried to yield the polymer product P2 (0.23 g, 96%).

**Polymer P3**

A mixture of polymer P2 (0.12 g), Phenylboron dichloride (0.1 mL), Triethylamine (5 drops) and 1,2-dichlorobenzene (10 mL) was heated at reflux and stirred under nitrogen for 3 h. The solution was then cooled to room temperature, precipitated in methanol, and filtered. The solids were washed three times with methanol and petroleum ether, respectively. Then dried to yield the final polymer product P3: browm soild (0.13 g, 92%)

**Polymer P4**

A mixture of compound M2 (0.075 g，0.086 mmol), 2,7-bis(4,4,5,5-tetramethyl-1,3,2-dioxaborolan-2-yl)-9-hexadecyl-9H-carbazole M3 (0.065 g，0.086 mmol), $NaHCO_3$ (0.35 g), THF (25 mL) and $H_2O$ (4 mL) was degassed, $Pd(PPh_3)_4$ (5 mg，0.0043 mmol) was added under a nitrogen atmosphere. The mixture was heated by microwave reactor at reflux and stirred under nitrogen for 4 h. The solution was then cooled to room temperature, precipitated in methanol, and filtered. The solids were washed three times with methanol and petroleum ether, respectively. Then dried to yield the polymer product P4 (0.091 g, 93%).

**Polymer P5**

A mixture of polymer P4 (0.08 g), NaOH (0.50 g), $H_2O$ (2 mL), $CH_3OH$ (3 mL) and THF (20 mL) were heated at reflux and stirred under nitrogen for 3 h. After cooling to the room temperature, the PH of solution was adjusted to 4~5. The solution was then cooled to room temperature, precipitated in methanol, and filtered. The solids were washed three times with methanol and petroleum ether, respectively. Then dried to yield the polymer product P5 (0.07 g, 96%).

**Polymer P6**

A mixture of polymer P5 (0.05 g), Phenylboron dichloride (0.1 mL), Triethylamine (5 drops) and 1,2-dichlorobenzene (10 mL) was heated at reflux and stirred under nitrogen for 3 h. The solution was then cooled to room temperature, precipitated in methanol, and filtered. The solids were washed three times with methanol and petroleum ether, respectively. Then dried to yield the final polymer product P6: browm soild (0.06 g, 95%).

**FTIR Spectra of Compounds**

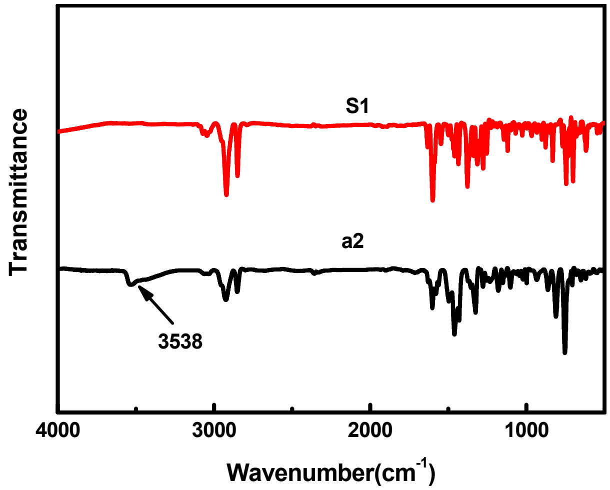


Figure S1. The FTIR spectra of compound a2 and S1

**Photophysical Properties of Compounds**

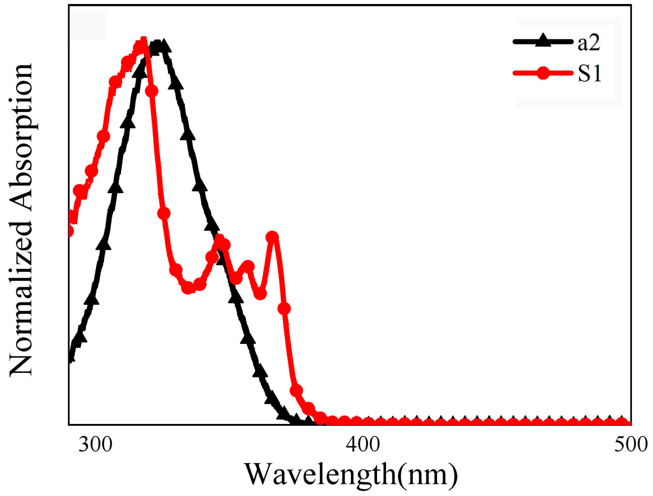


Figure S2. The UV-vis absorption spectra of compound a2 and S1

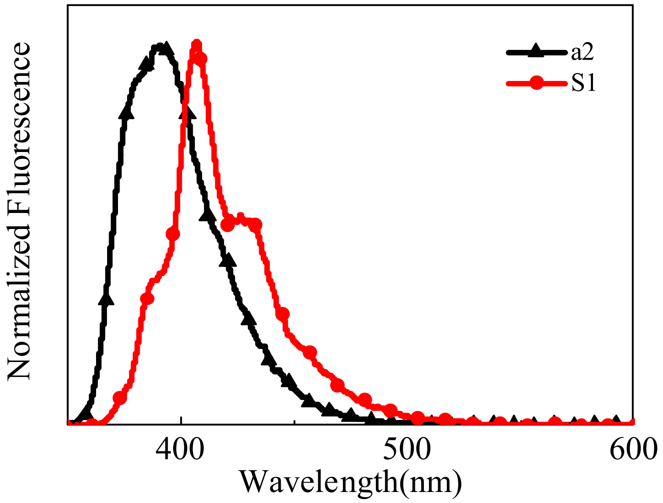


Figure S3. The fluorescence emission spectra of compound a2 and S1

## Electrochemical Properties of Compounds and Polymers

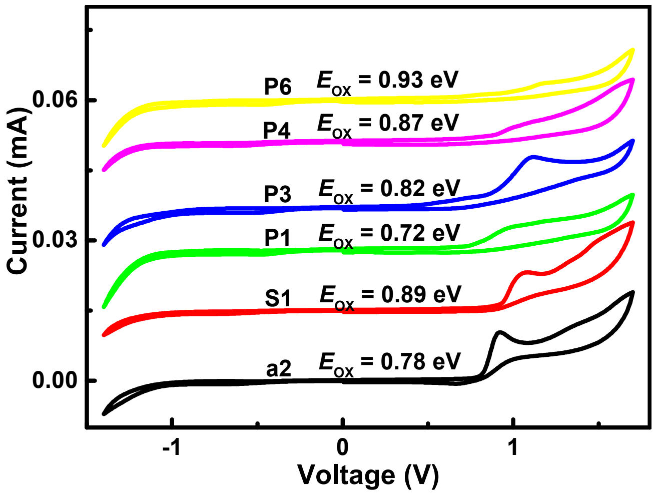


Figure S4. The cyclic voltammograms of compounds and polymers

Table S1. the CV date of compounds and polymers

| | HOMO/eV | LUMO/eV | Band gap/eV |
|---|---|---|---|
| a2 | -5.58 | -2.19 | 3.39 |
| S1 | -5.69 | -2.39 | 3.30 |
| P1 | -5.52 | -2.38 | 3.14 |
| P3 | -5.62 | -2.76 | 2.86 |
| P4 | -5.60 | -2.60 | 3.00 |
| P6 | -5.68 | -3.02 | 2.66 |

## The NMR spectra and MALDI-TOF spectra

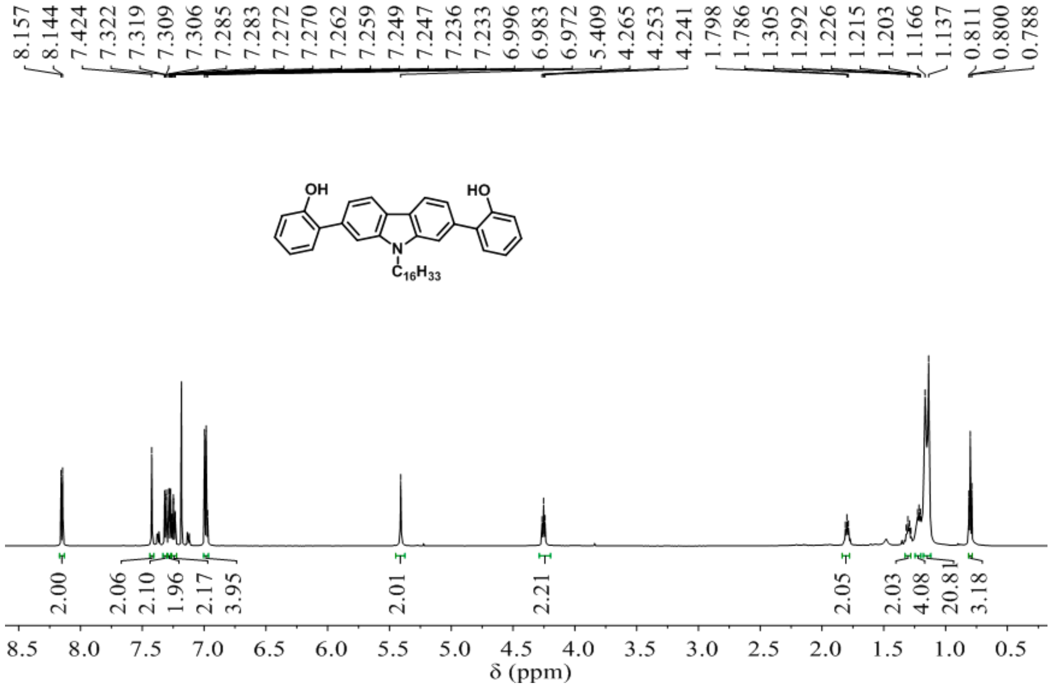

Figure S5. $^{1}H$-NMR of the compound a2 recorded in $CDCl_3$.

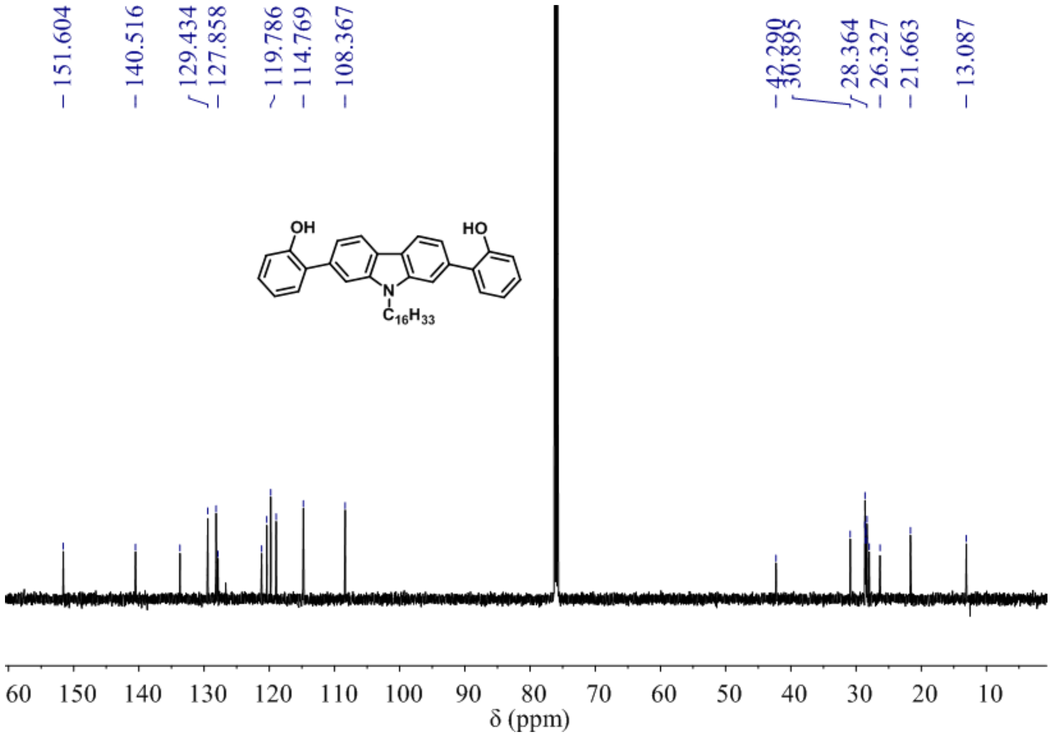

Figure S6. $^{13}C$-NMR of the compound a2 recorded in $CDCl_3$.

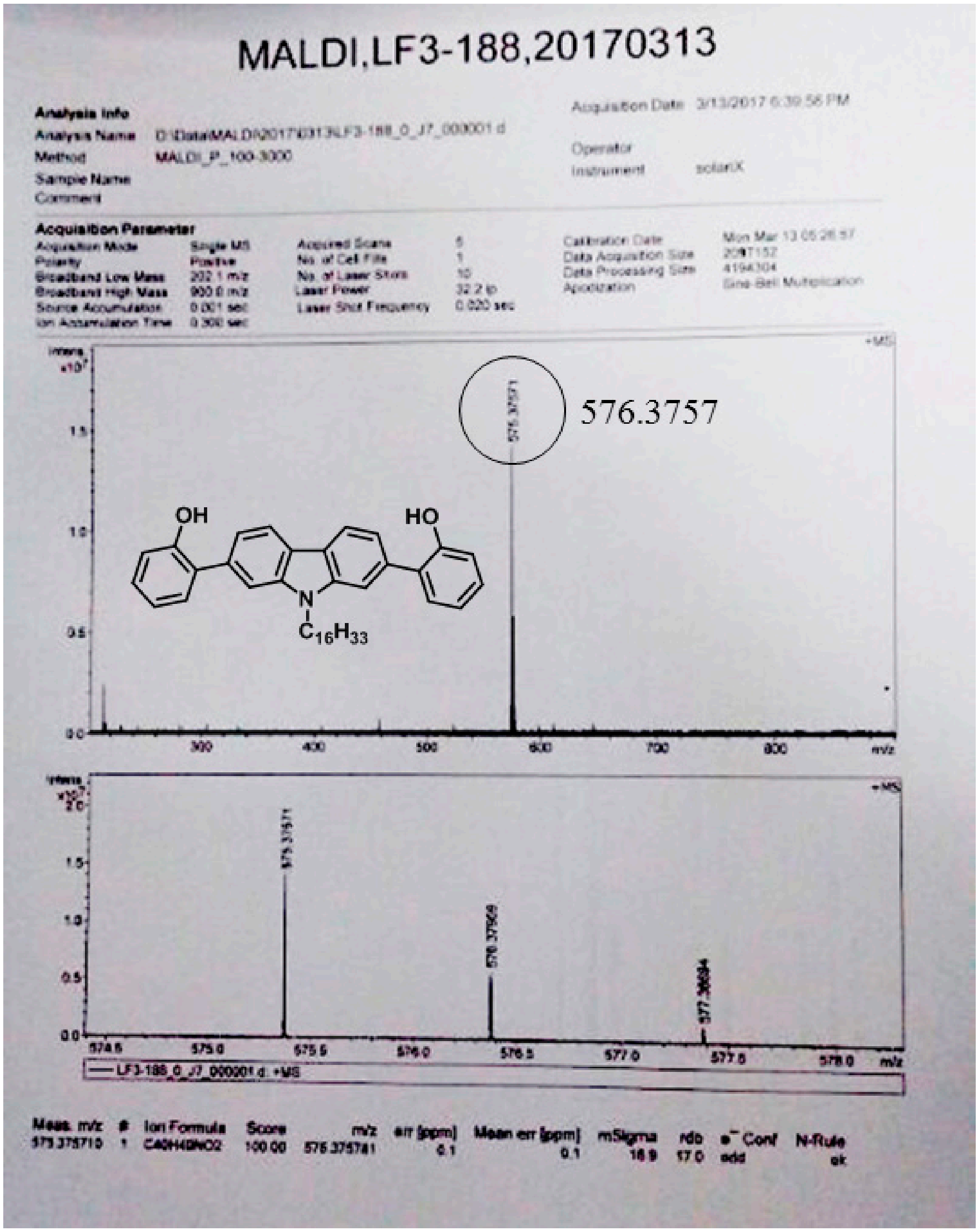


Figure S7. High resolution MALDI-TOF spectra of the a2.

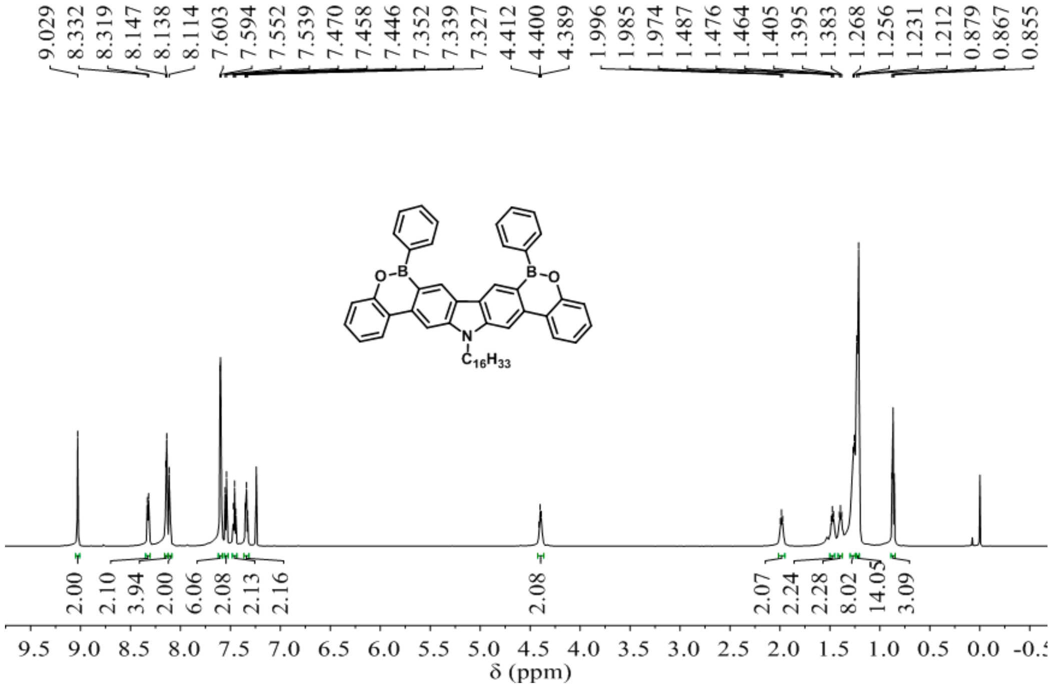


Figure S8. $^1$H-NMR of the compound S1 recorded in $CDCl_3$.

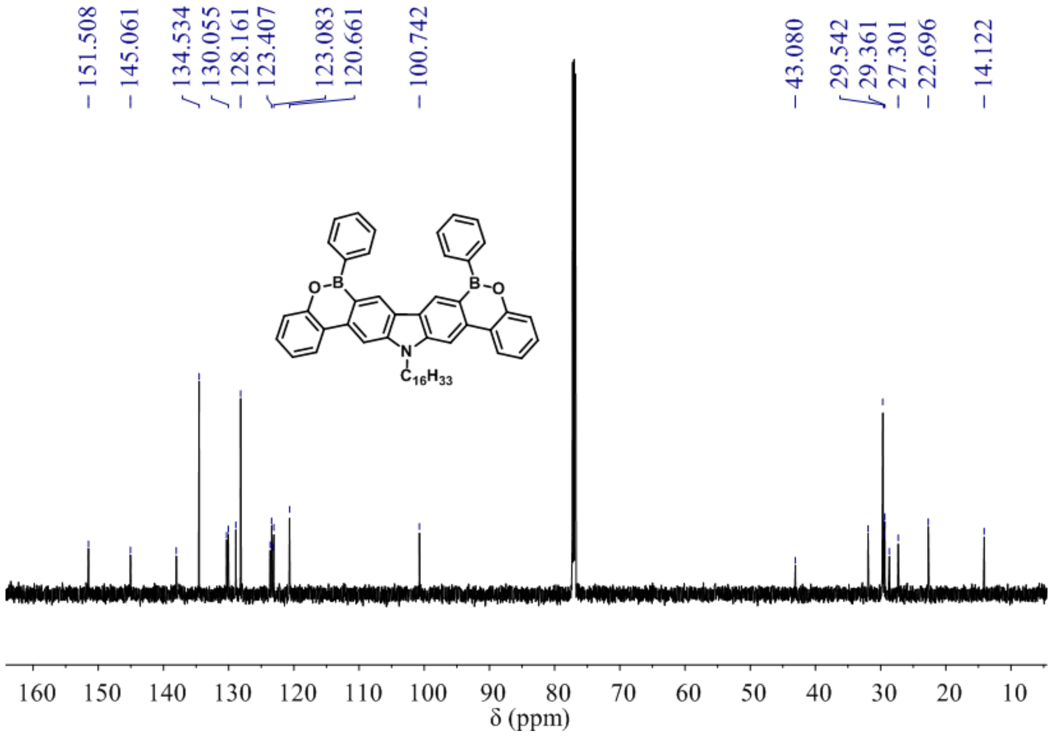


Figure S9. $^{13}$C-NMR of the compound S1 recorded in $CDCl_3$.

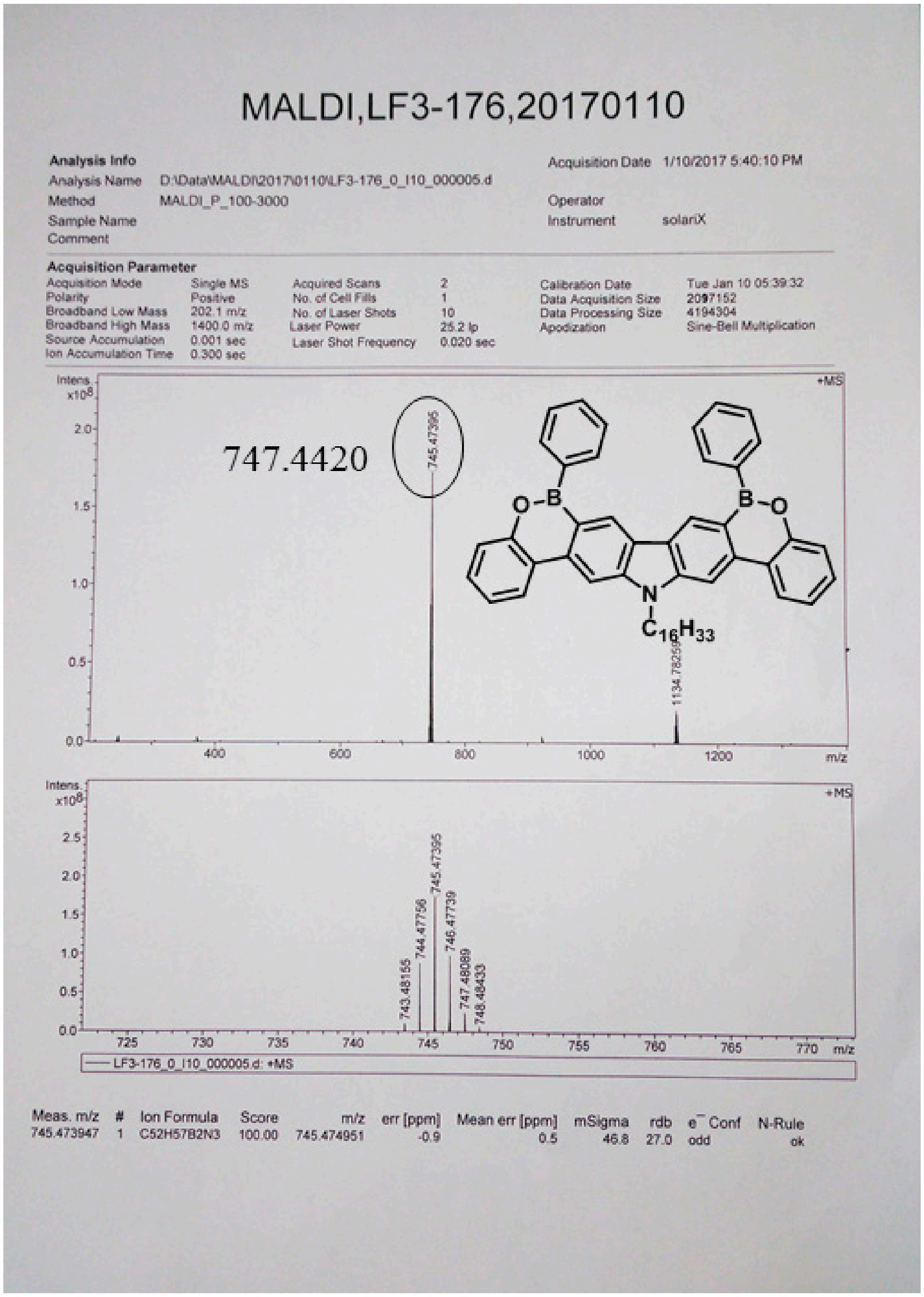


Figure S10. High resolution MALDI-TOF spectra of the S1.

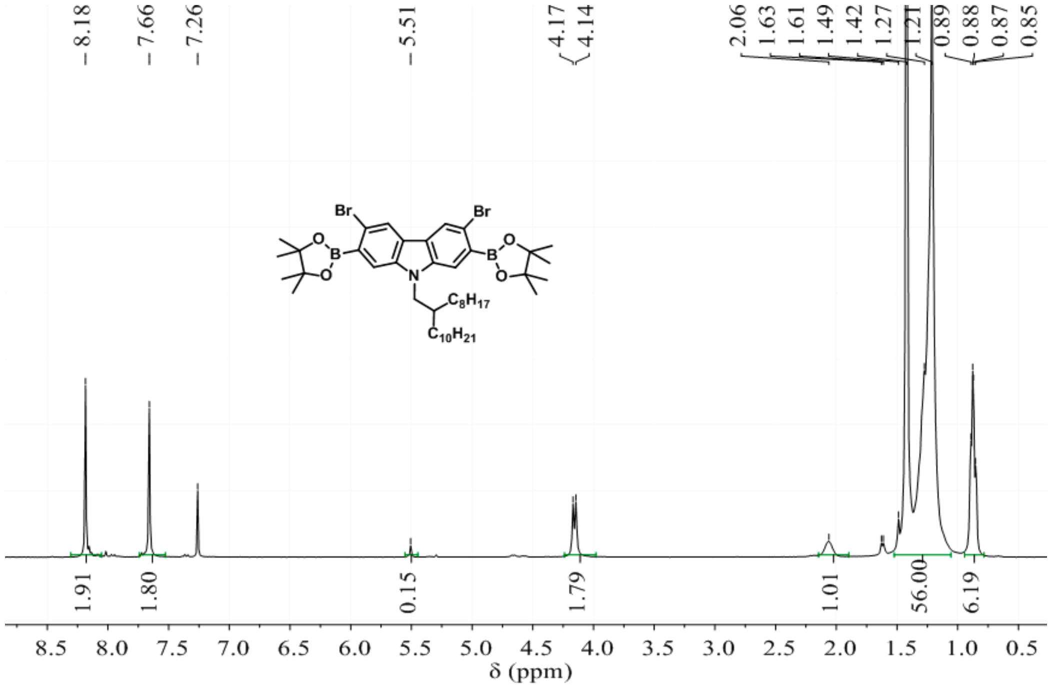


Figure S11. $^{1}$H-NMR of the compound a4 recorded in $CDCl_3$.

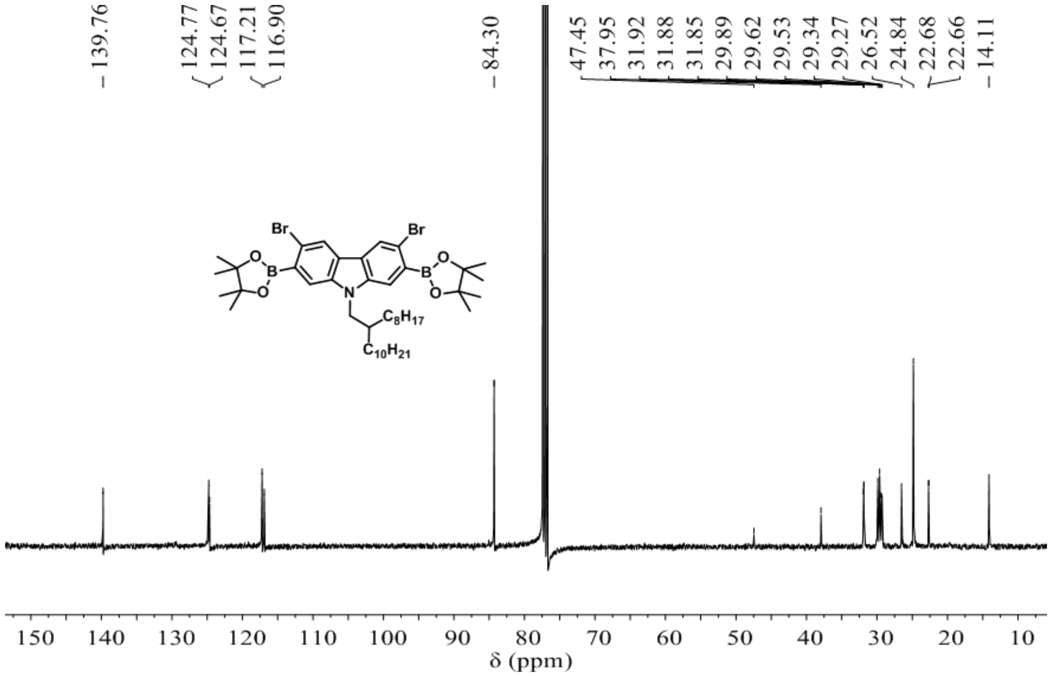


Figure S12. $^{13}$C-NMR of the compound a4 recorded in $CDCl_3$.

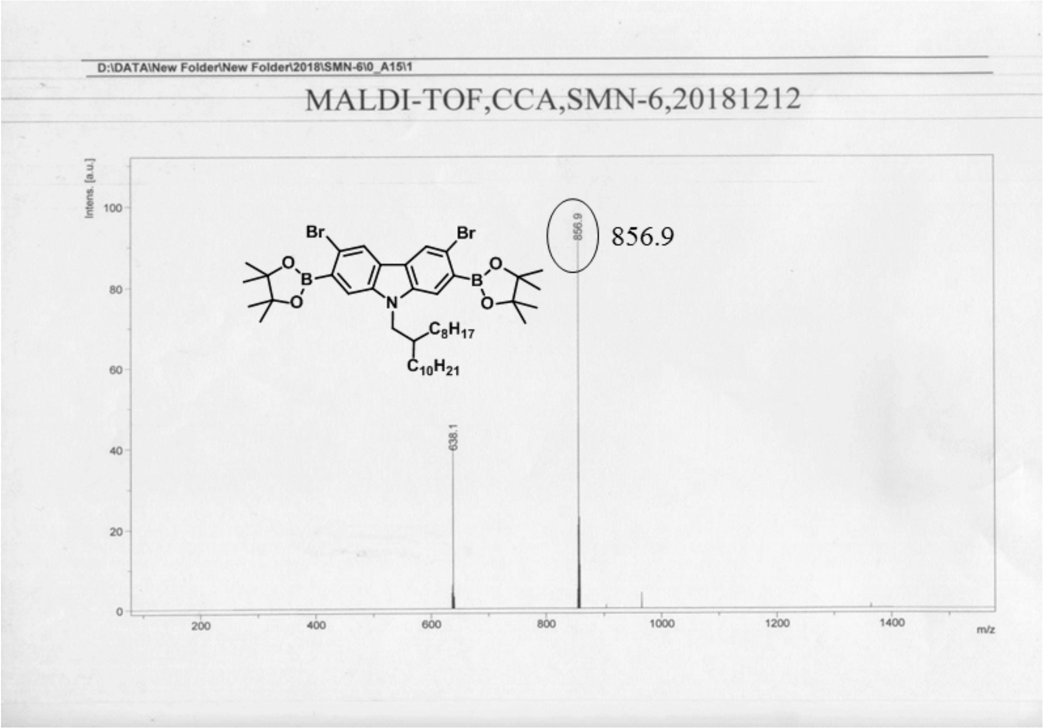


Figure S13. High resolution MALDI-TOF spectra of the a4.

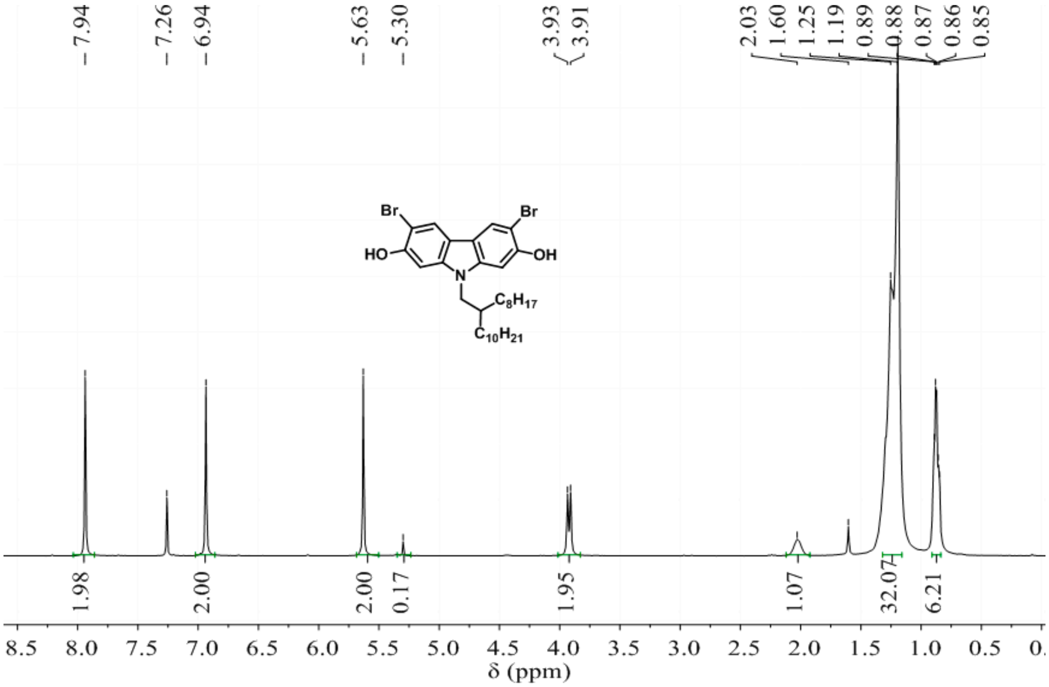


Figure S14. $^{1}$H-NMR of the compound a5 recorded in $CDCl_3$.

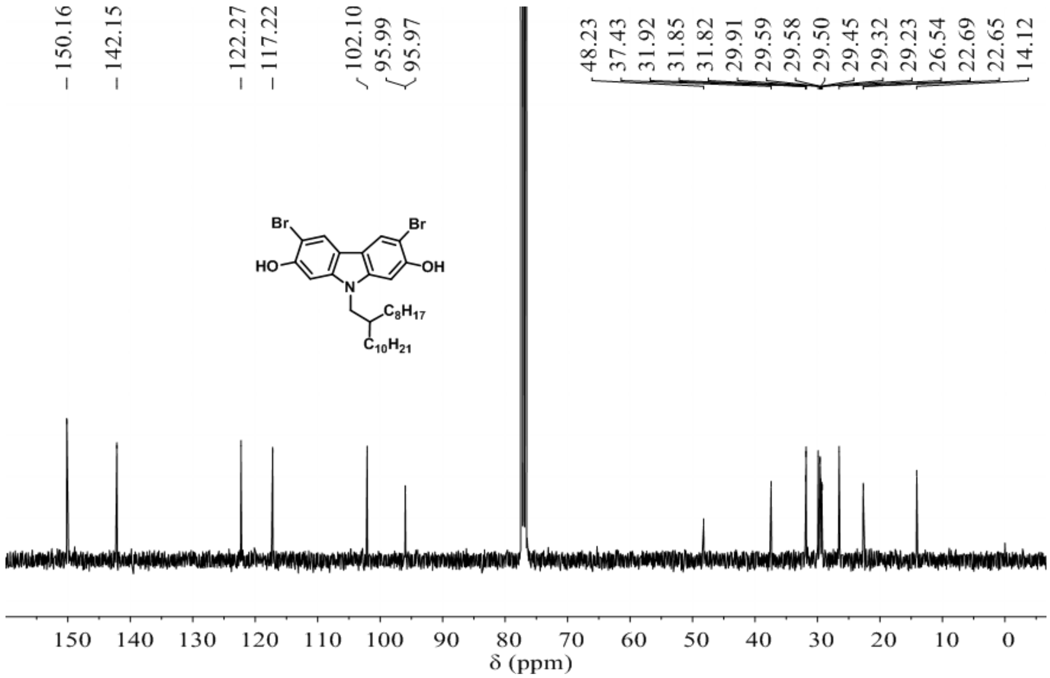


Figure S15. $^{13}$C-NMR of the compound a5 recorded in $CDCl_3$.

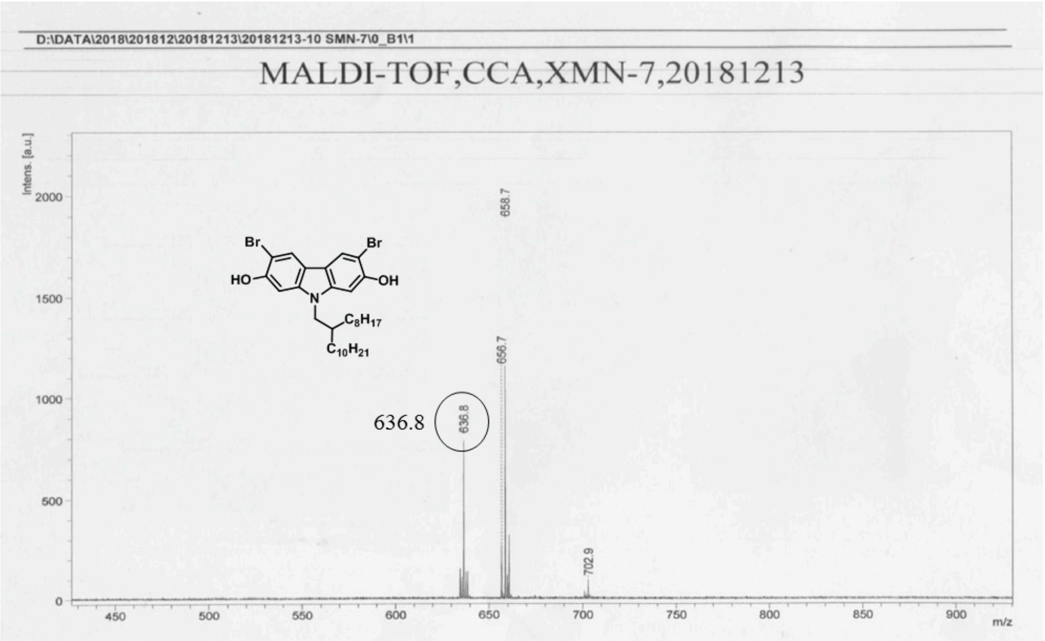


Figure S16. High resolution MALDI-TOF spectra of the a5.

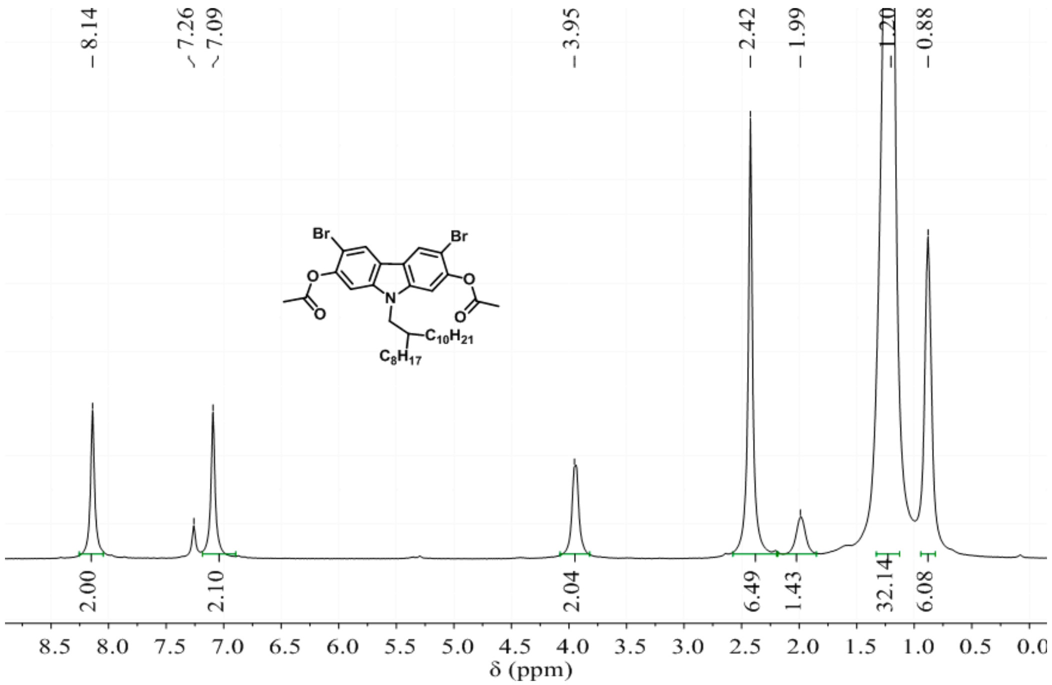


Figure S17. $^{1}$H-NMR of the compound M1 recorded in $CDCl_3$.

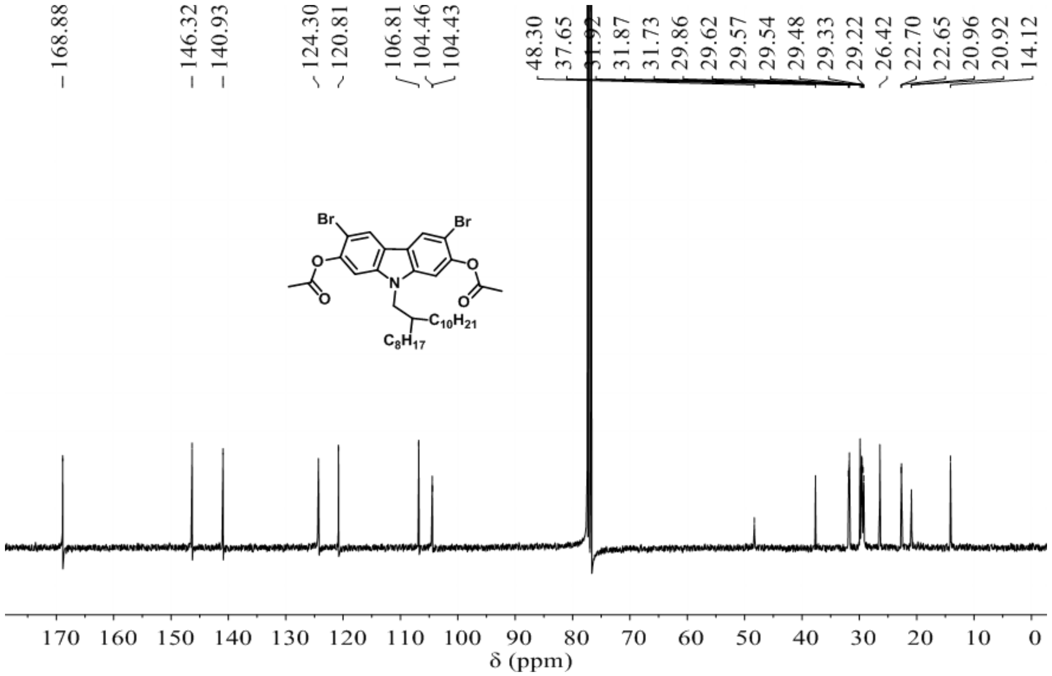


Figure S18. $^{13}$C-NMR of the compound M1 recorded in $CDCl_3$.

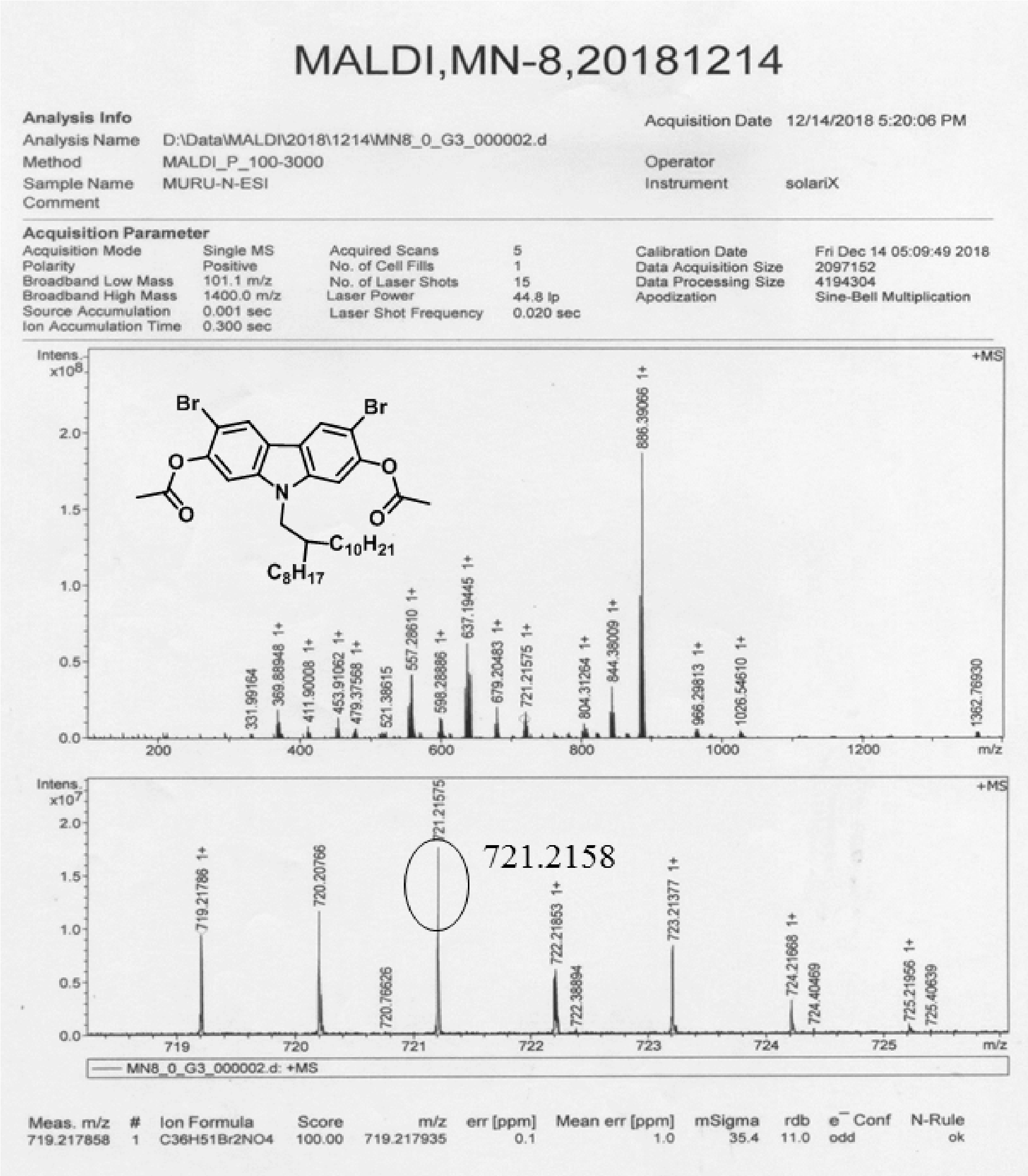

# MALDI,MN-8,20181214

**Analysis Info**

| | | | |
|---|---|---|---|
| Analysis Name | D:\Data\MALDI\2018\1214\MN8_0_G3_000002.d | Acquisition Date | 12/14/2018 5:20:06 PM |
| Method | MALDI_P_100-3000 | Operator | |
| Sample Name | MURU-N-ESI | Instrument | solariX |
| Comment | | | |

**Acquisition Parameter**

| | | | | | |
|---|---|---|---|---|---|
| Acquisition Mode | Single MS | Acquired Scans | 5 | Calibration Date | Fri Dec 14 05:09:49 2018 |
| Polarity | Positive | No. of Cell Fills | 1 | Data Acquisition Size | 2097152 |
| Broadband Low Mass | 101.1 m/z | No. of Laser Shots | 15 | Data Processing Size | 4194304 |
| Broadband High Mass | 1400.0 m/z | Laser Power | 44.8 lp | Apodization | Sine-Bell Multiplication |
| Source Accumulation | 0.001 sec | Laser Shot Frequency | 0.020 sec | | |
| Ion Accumulation Time | 0.300 sec | | | | |



| Meas. m/z | # | Ion Formula | Score | m/z | err [ppm] | Mean err [ppm] | mSigma | rdb | e⁻ Conf | N-Rule |
|---|---|---|---|---|---|---|---|---|---|---|
| 719.217858 | 1 | C36H51Br2NO4 | 100.00 | 719.217935 | 0.1 | 1.0 | 35.4 | 11.0 | odd | ok |

Figure S19. High resolution MALDI-TOF spectra of the M1.

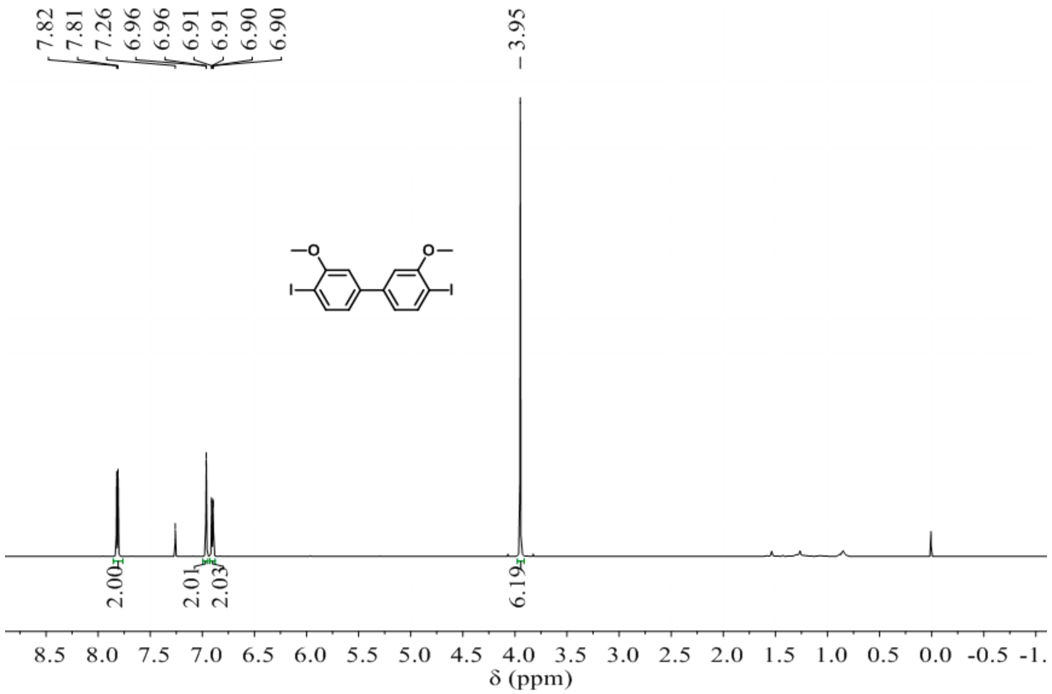


Figure S20. $^{1}$H-NMR of the compound a7 recorded in $CDCl_3$.

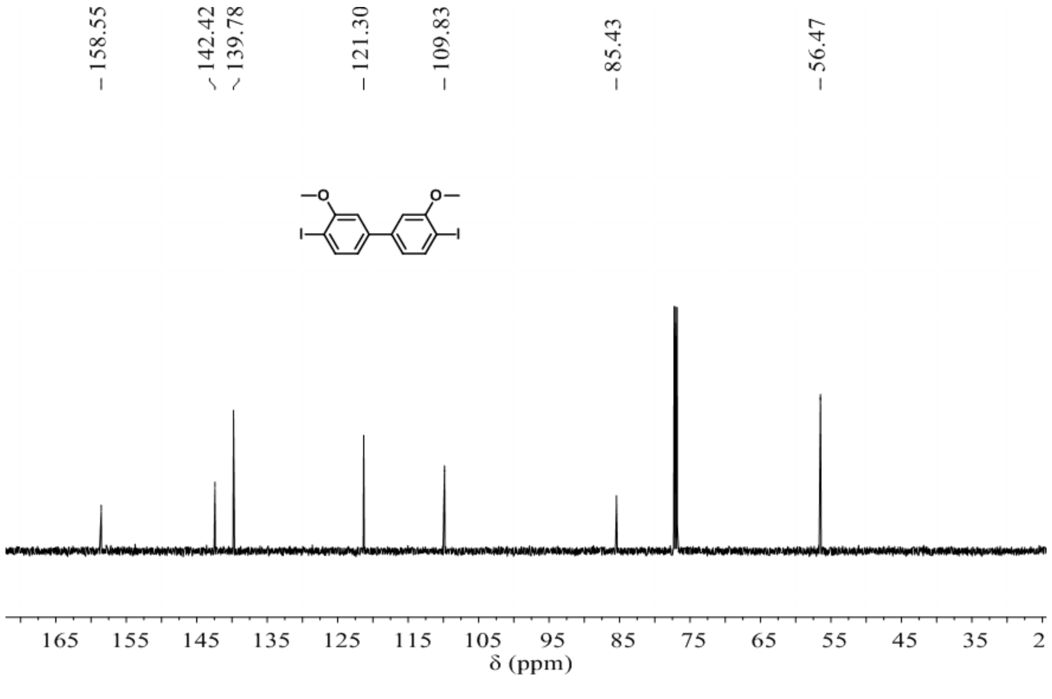


Figure S21. $^{13}$C-NMR of the compound a7 recorded in $CDCl_3$.

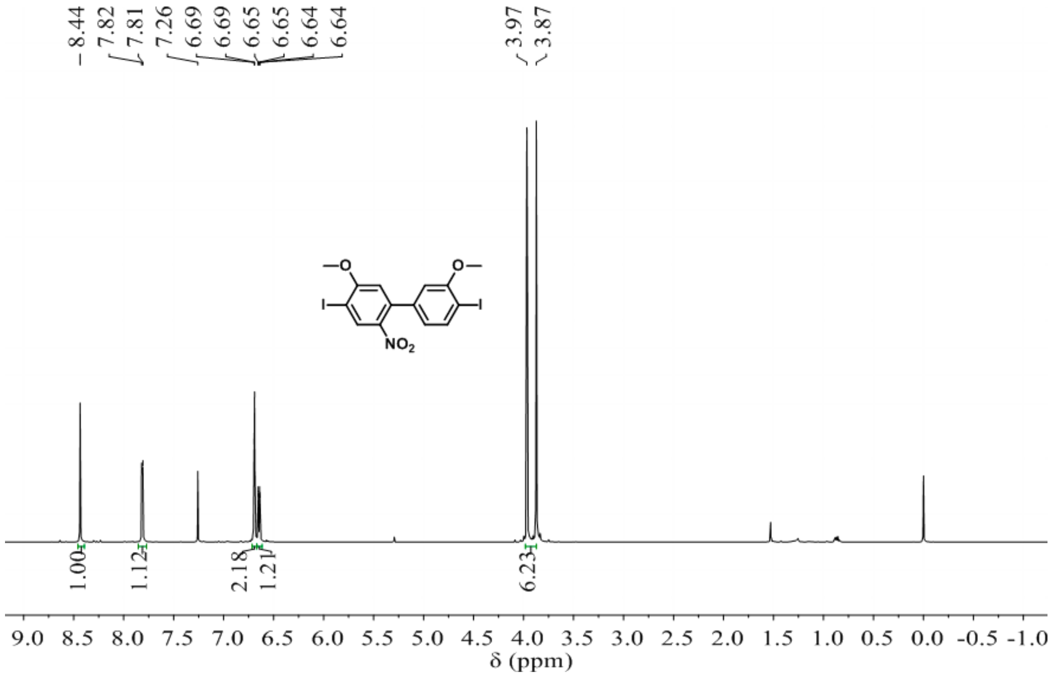


Figure S22. $^{1}$H-NMR of the compound a8 recorded in $CDCl_3$.

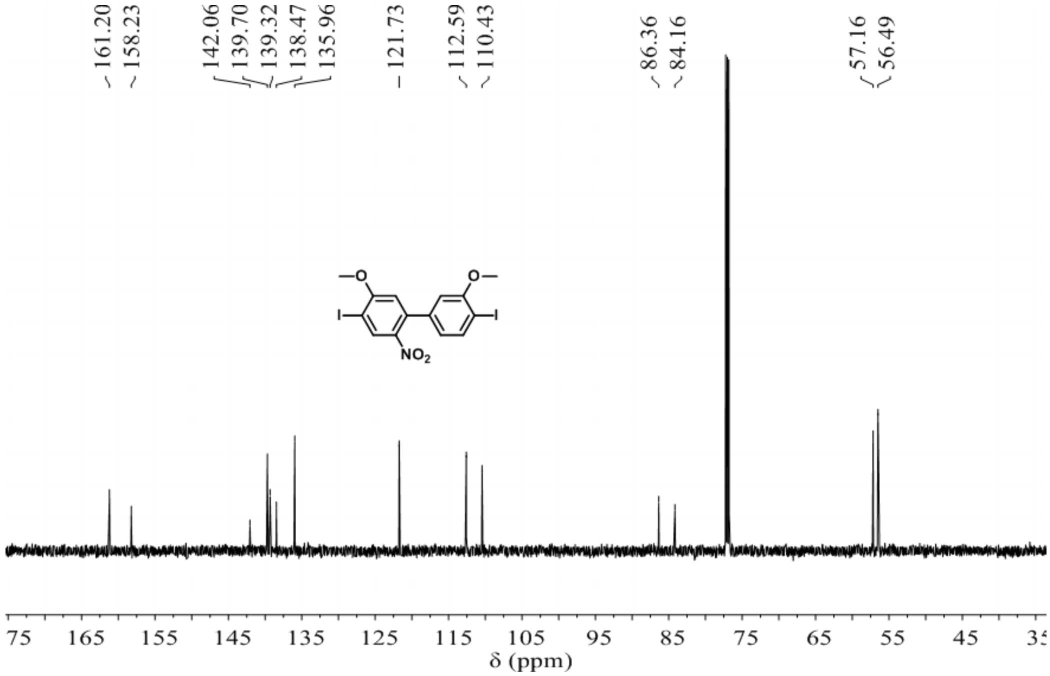


Figure S23. $^{13}$C-NMR of the compound a8 recorded in $CDCl_3$.

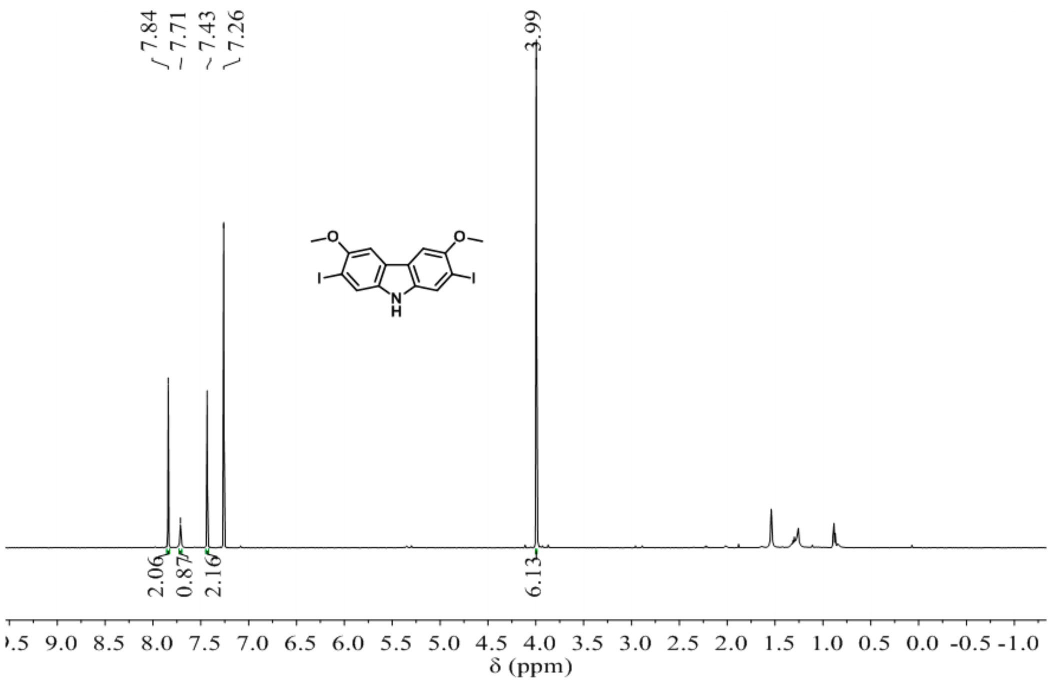


Figure S24. $^{1}$H-NMR of the compound a9 recorded in $CDCl_3$.

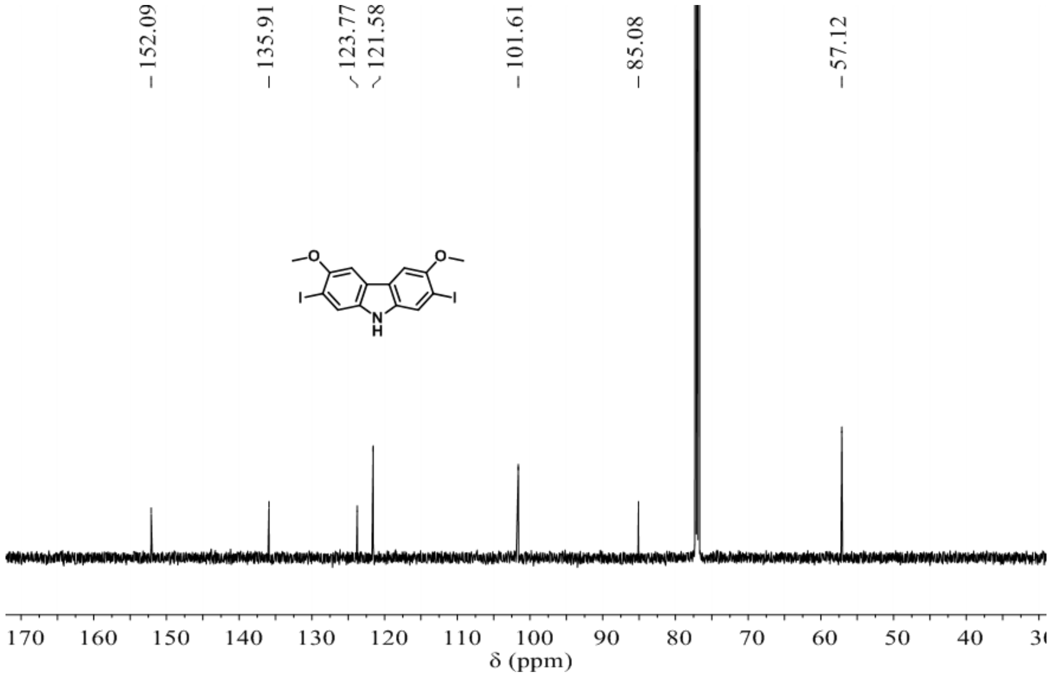


Figure S25. $^{13}$C-NMR of the compound a9 recorded in $CDCl_3$.

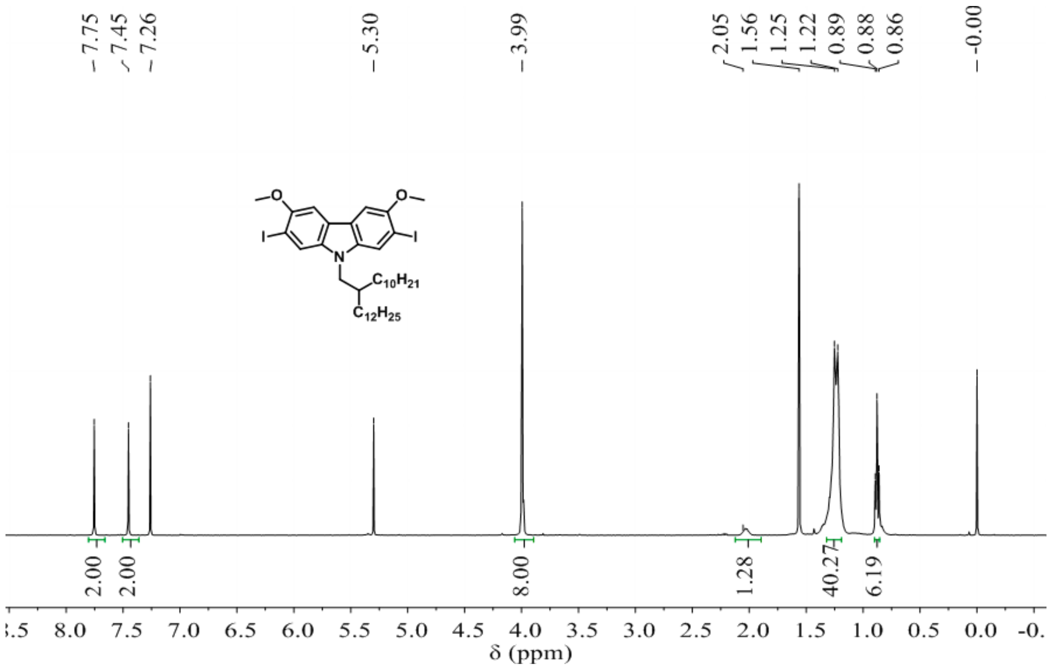


Figure S26. $^{1}$H-NMR of the compound a10 recorded in $CDCl_3$.

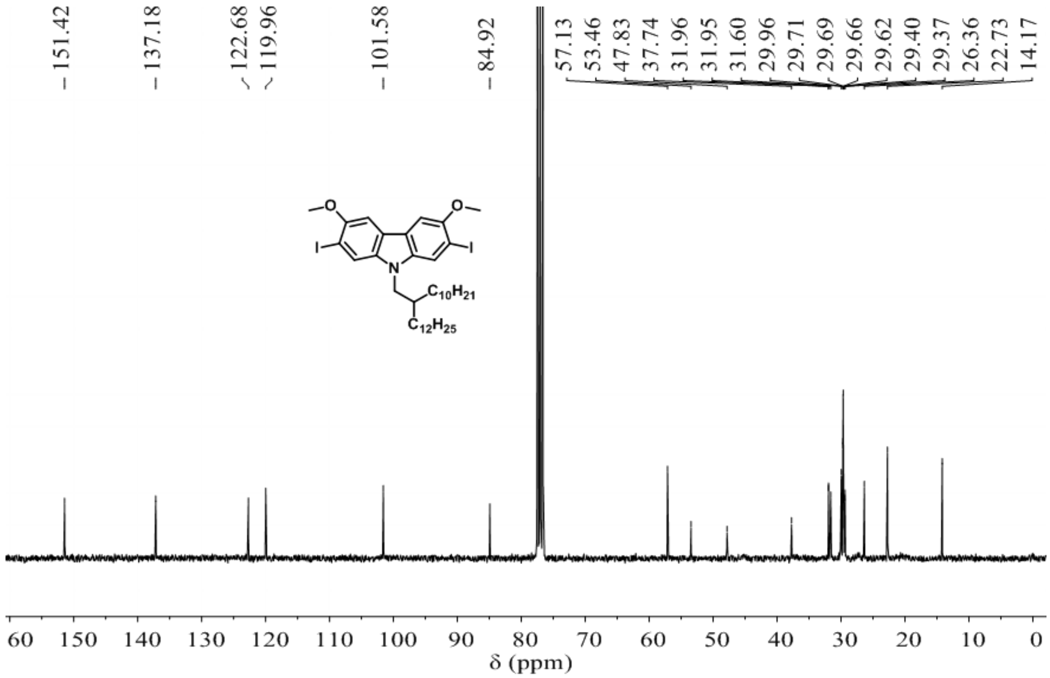


Figure S27. $^{13}$C-NMR of the compound a10 recorded in $CDCl_3$.

# MALDI,S-9,20190118

**Analysis Info**

Acquisition D 1/18/2019 4:27:25 PM

Analysis Name D:\Data\MALDI\2019\0118\S-9_0_H4_000001.d
Method MALDI_P_100-3000
Sample Name MURU-N-ESI
Comment

Operator
Instrument solariX

**Acquisition Paramet**

| | | | | | |
|---|---|---|---|---|---|
| Acquisition Mode | Single MS | Acquired Scans | 3 | Calibration Date | Wed Jan 16 05:33:43 2019 |
| Polarity | Positive | No. of Cell Fills | 1 | Data Acquisition Size | 2097152 |
| Broadband Low Mass | 202.1 m/z | No. of Laser Shots | 10 | Data Processing Size | 4194304 |
| Broadband High Mass | 1600.0 m/z | Laser Power | 50.4 lp | Apodization | Sine-Bell Multiplication |
| Source Accumulation | 0.001 sec | Laser Shot Frequency | 0.020 sec | | |
| Ion Accumulation Time | 0.300 sec | | | | |

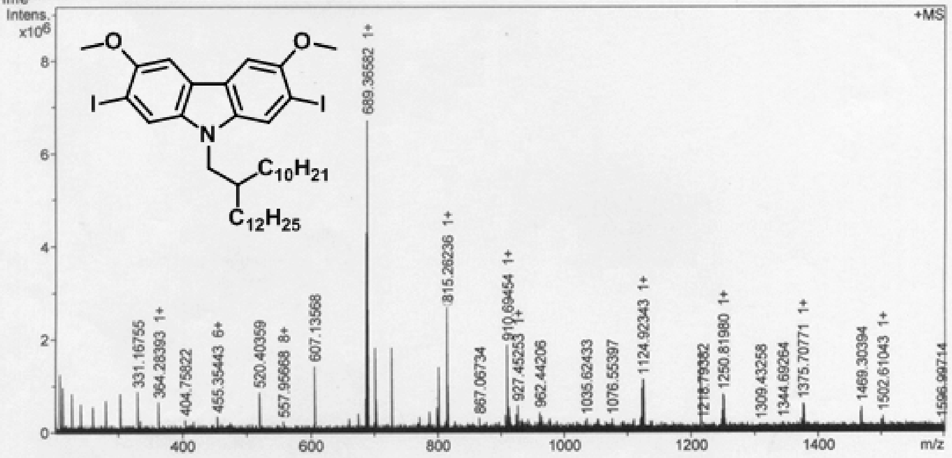


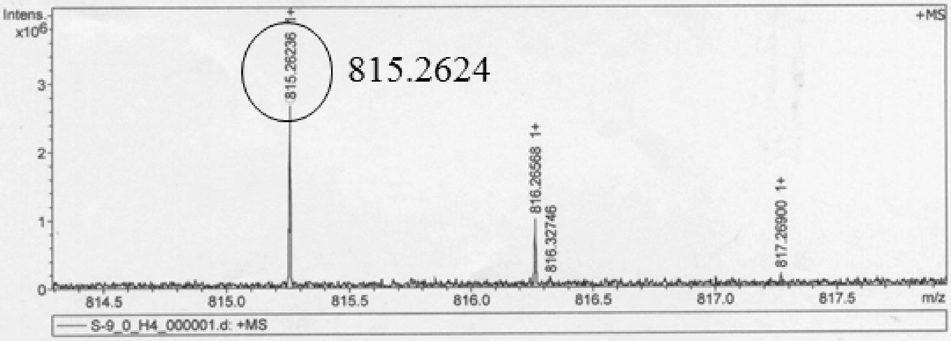


| Meas. m/z | # | Ion Formula | Score | m/z | err [ppm] | Mean err [ppm] | mSigma | rdb | e⁻ Conf | N-Rule |
|---|---|---|---|---|---|---|---|---|---|---|
| 815.262356 | 1 | C38H59I2NO2 | 100.00 | 815.262978 | 0.8 | 0.8 | 25.7 | 9.0 | odd | ok |

Figure S28. High resolution MALDI-TOF spectra of the a10.

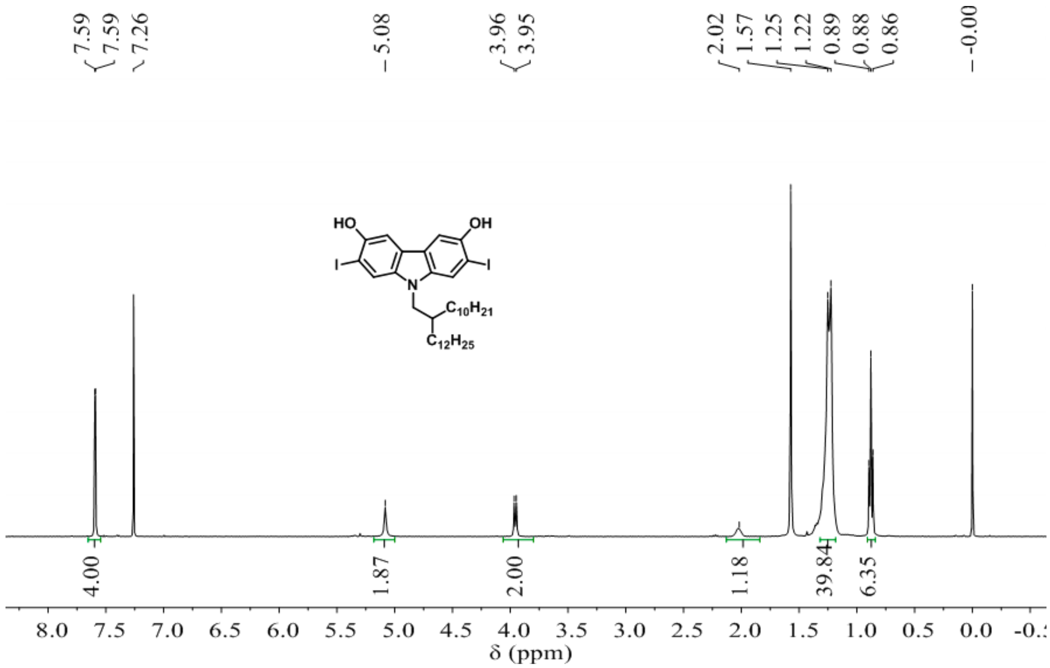


Figure S29. $^{1}$H-NMR of the compound a11 recorded in $CDCl_3$.

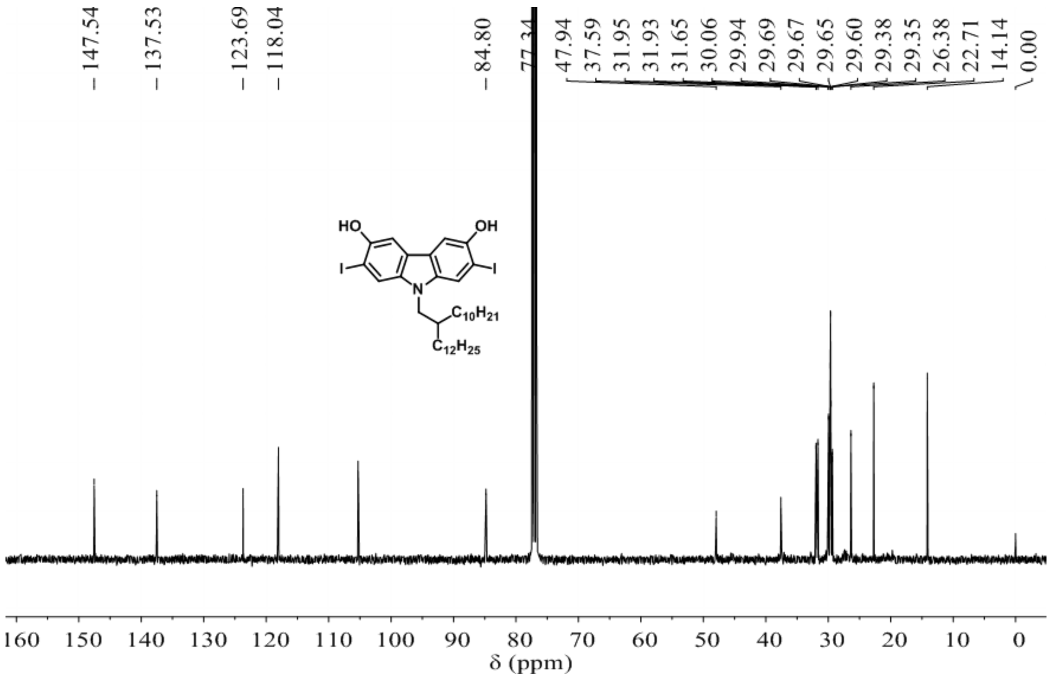


Figure S30. $^{13}$C-NMR of the compound a11 recorded in $CDCl_3$.

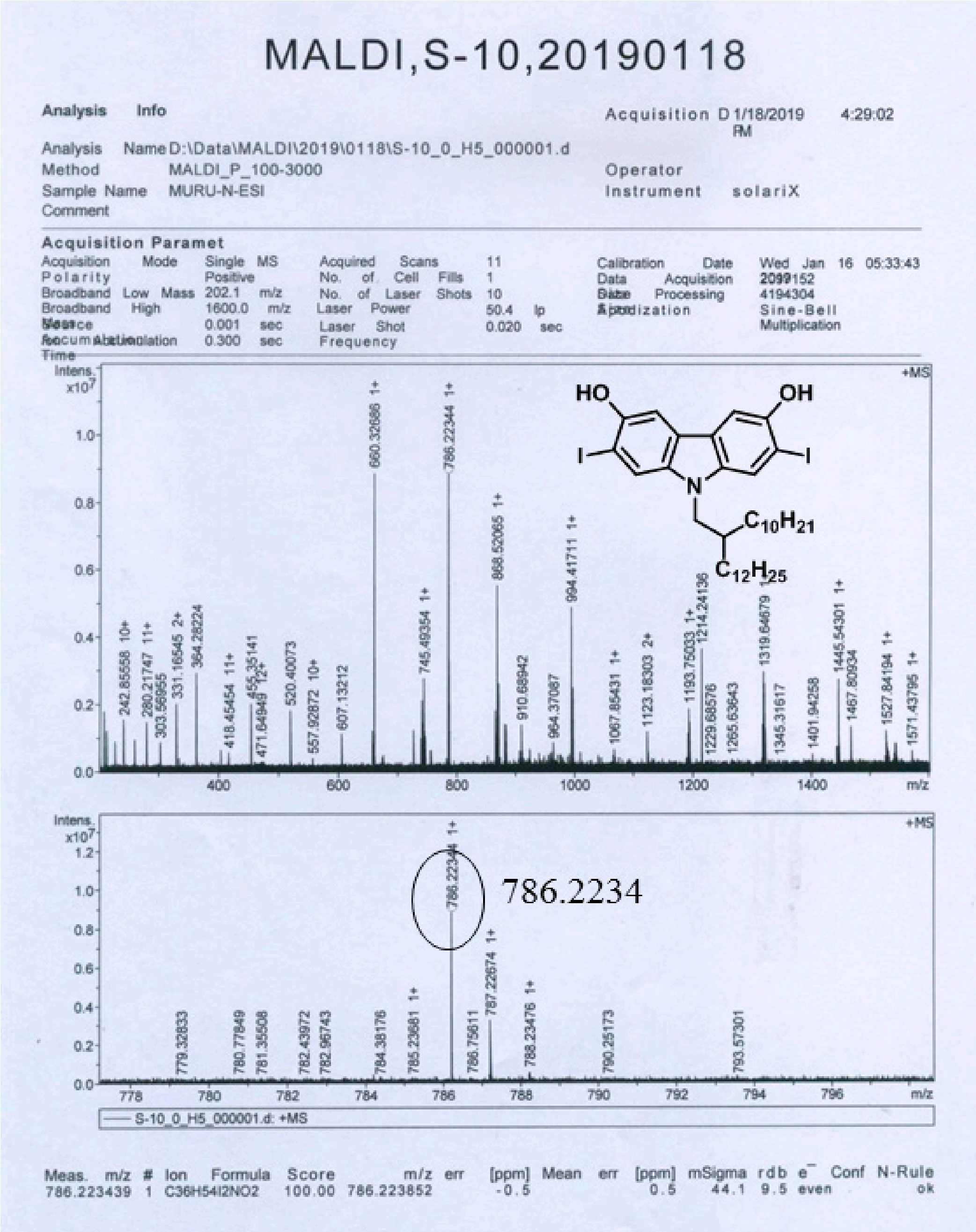


Figure S31. High resolution MALDI-TOF spectra of the a11.

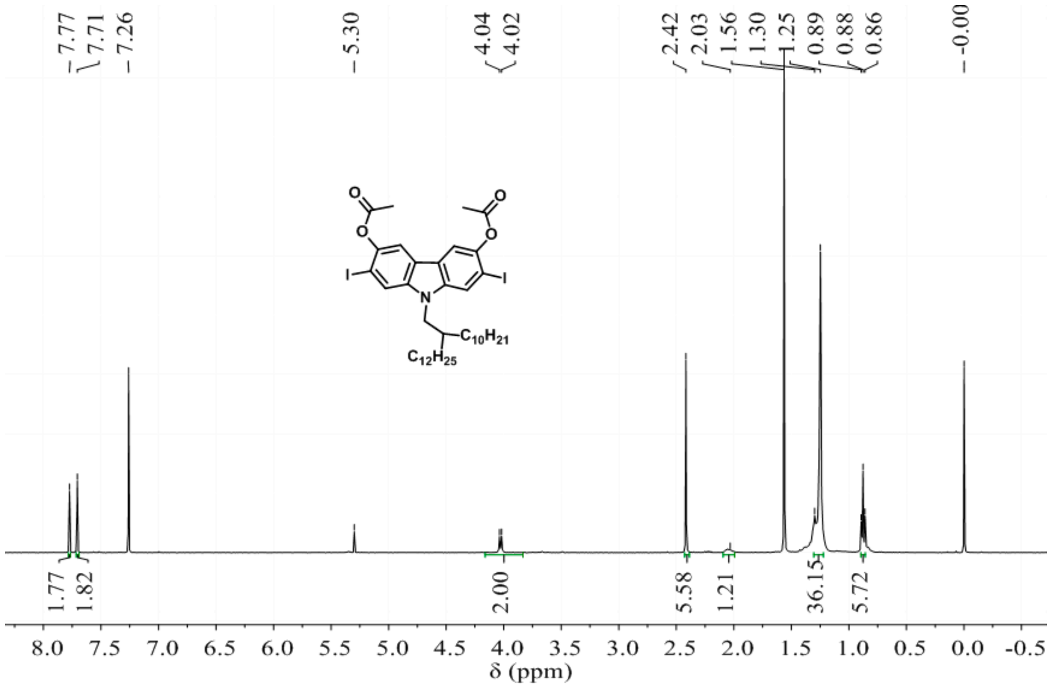


Figure S32. $^{1}$H-NMR of the compound M2 recorded in $CDCl_3$.

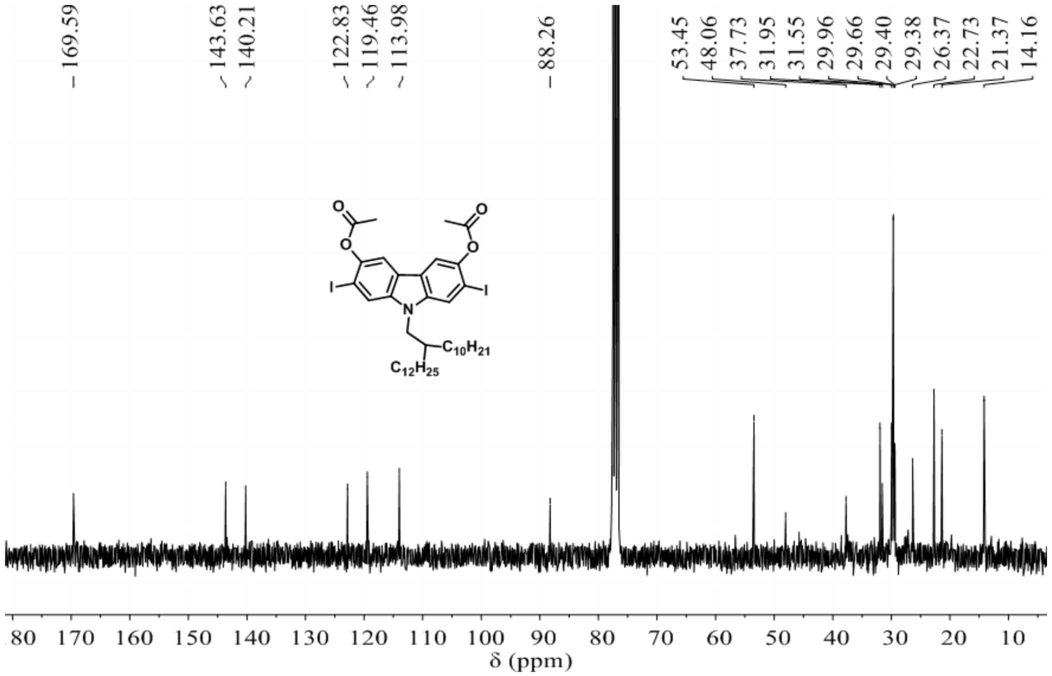


Figure S33. $^{13}$C-NMR of the compound M2 recorded in $CDCl_3$.

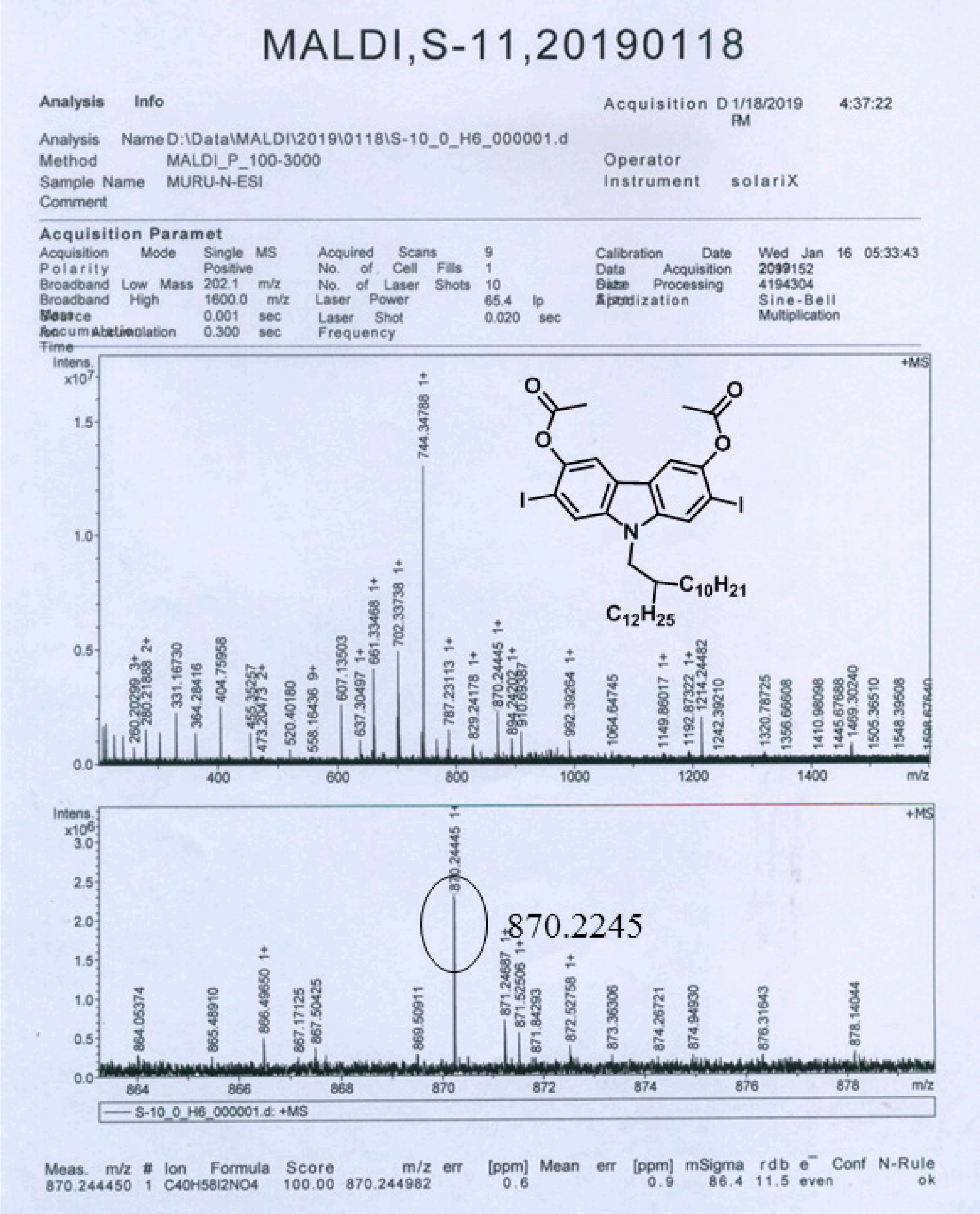


Figure S34. High resolution MALDI-TOF spectra of the M2.

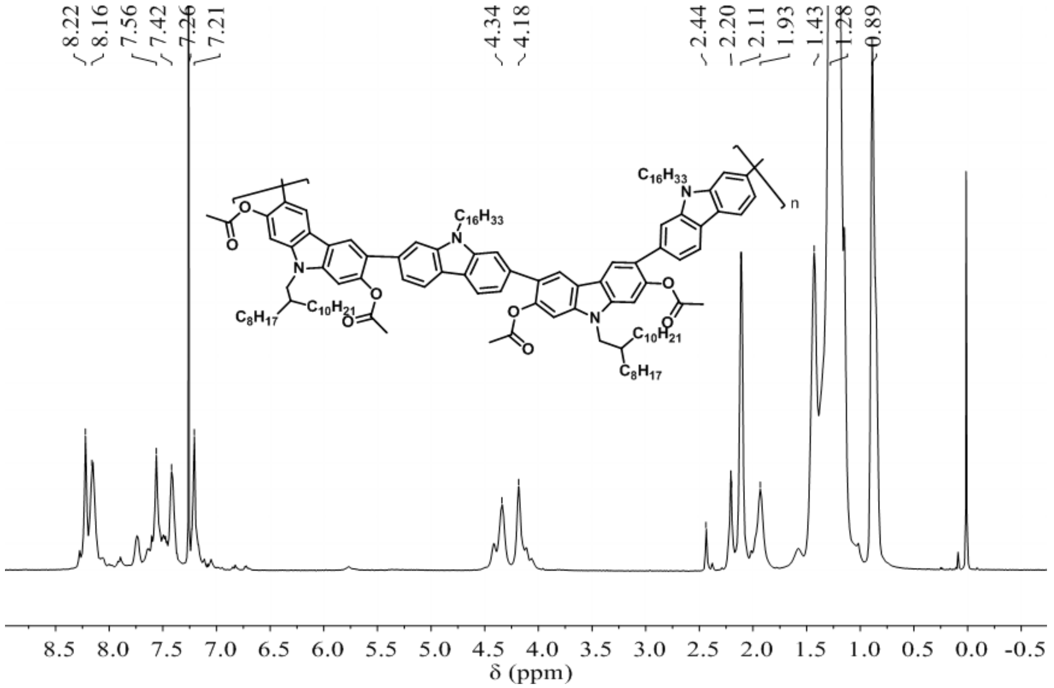

Figure S35. $^{1}$H-NMR of the compound P1 recorded in $CDCl_3$.

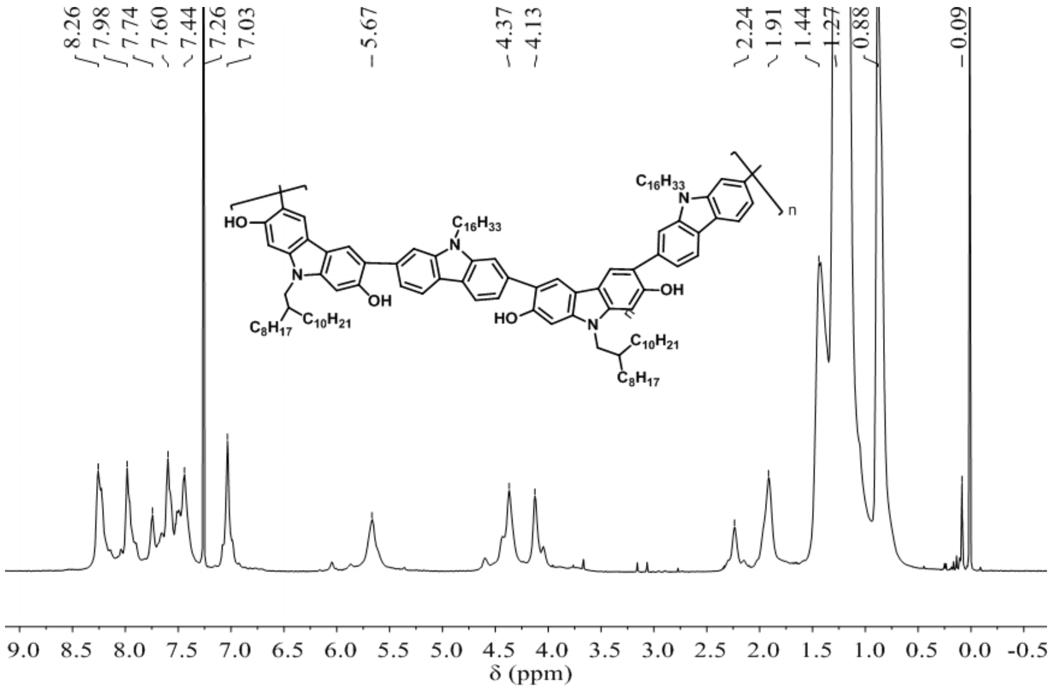

Figure S36. $^{1}$H-NMR of the compound P2 recorded in $CDCl_3$.

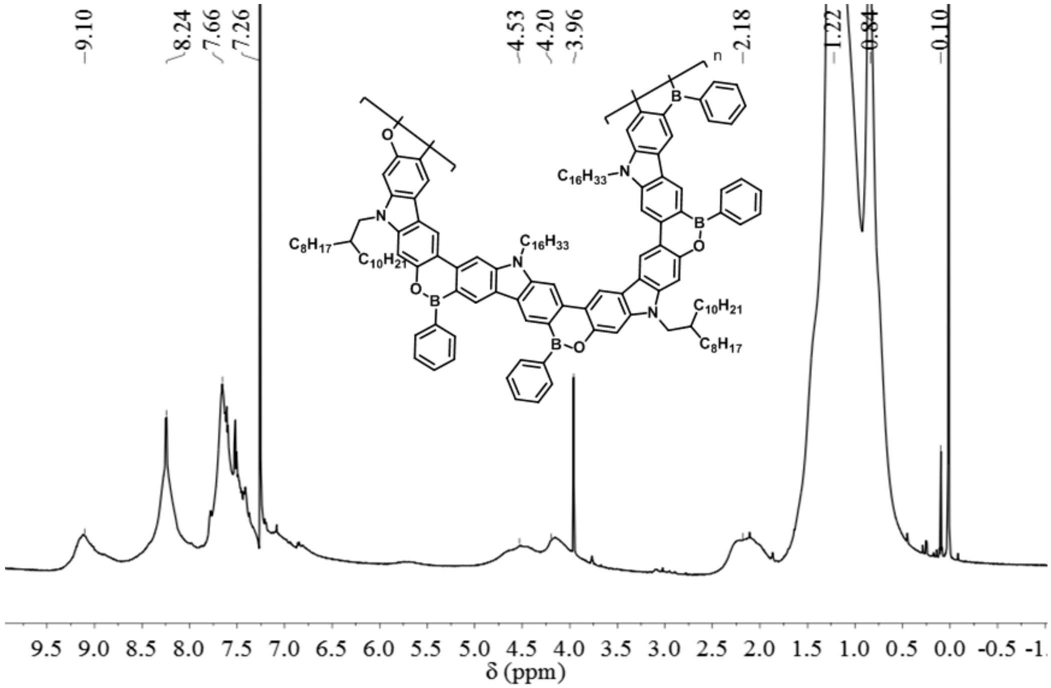


Figure S37. $^{1}$H-NMR of the compound P3 recorded in $CDCl_3$.

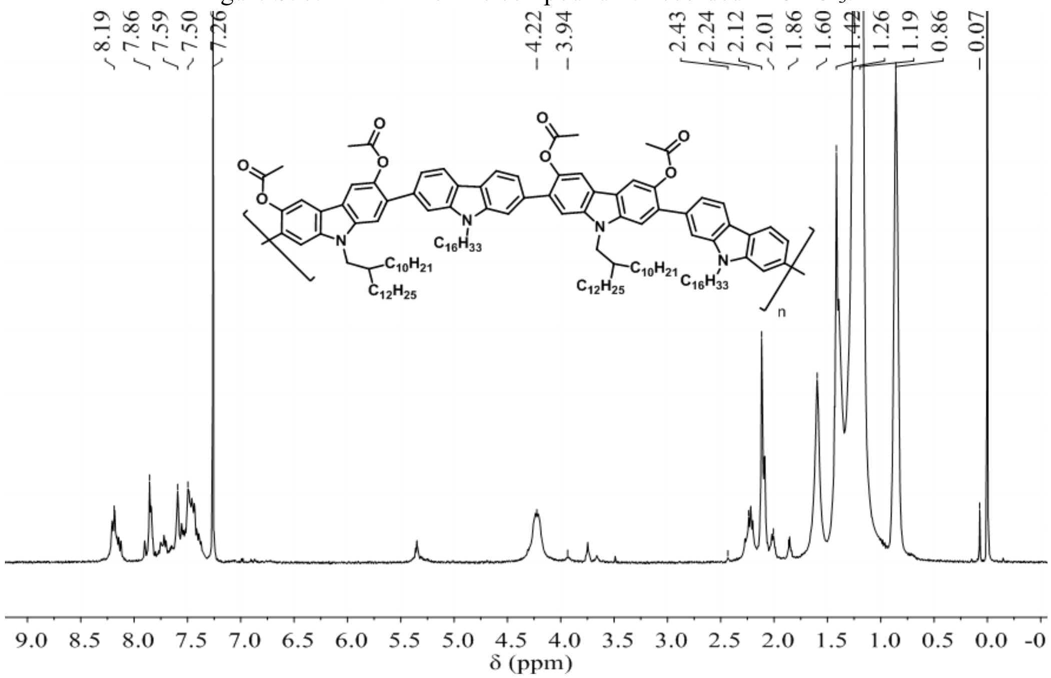


Figure S38. $^{1}$H-NMR of the compound P4 recorded in $CDCl_3$.

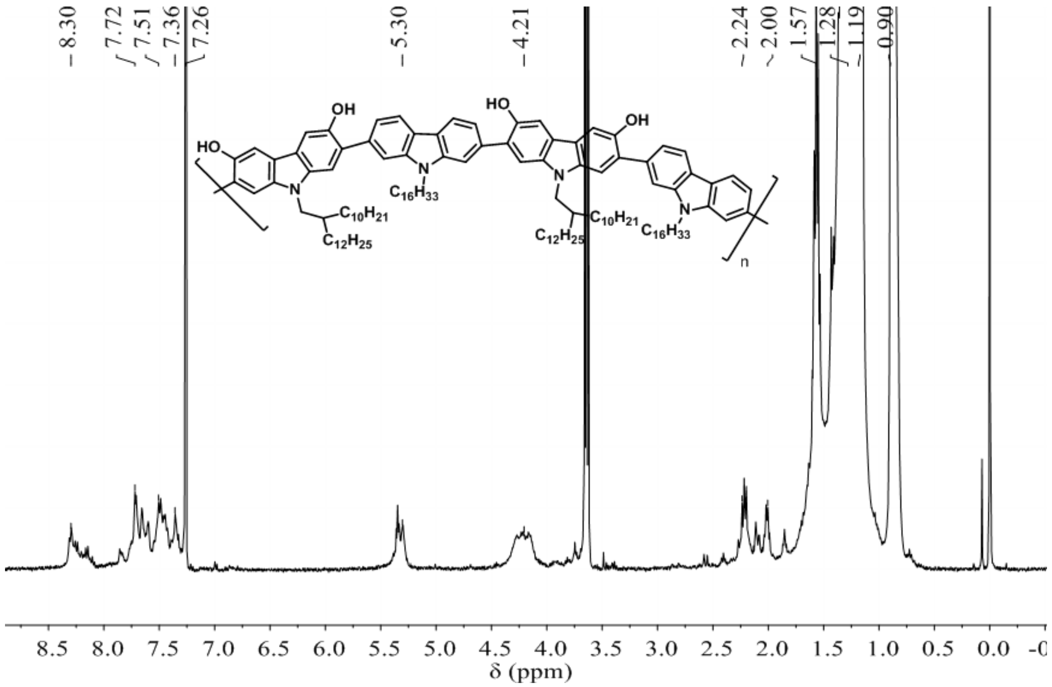


Figure S39. $^{1}$H-NMR of the compound P5 recorded in $CDCl_3$.

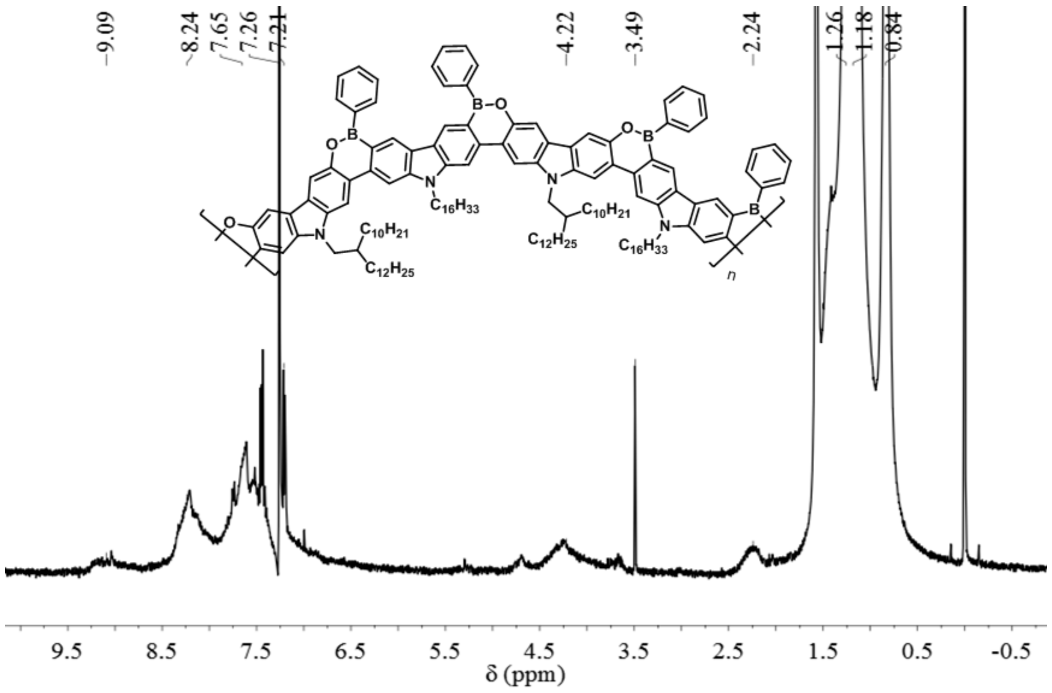


Figure S40. $^{1}$H-NMR of the compound P6 recorded in $CDCl_3$.
